# A Drug Screening Revealed Novel Potential Agents against Malignant Pleural Mesothelioma

**DOI:** 10.3390/cancers14102527

**Published:** 2022-05-20

**Authors:** Irene Dell’Anno, Alessandra Melani, Sarah A. Martin, Marcella Barbarino, Roberto Silvestri, Monica Cipollini, Antonio Giordano, Luciano Mutti, Andrea Nicolini, Luca Luzzi, Raffaele Aiello, Federica Gemignani, Stefano Landi

**Affiliations:** 1 Genetic Unit, Department of Biology, University of Pisa, 56126 Pisa, Italy; irene.dellanno@biologia.unipi.it (I.D.); a.melani4@studenti.unipi.it (A.M.); roberto.silvestri@biologia.unipi.it (R.S.); monica.cipollini@unipi.it (M.C.); stefano.landi@unipi.it (S.L.); 2 Centre for Cancer Cell and Molecular Biology, Barts Cancer Institute, Queen Mary University of London, Charterhouse Square, London EC1M 6BQ, UK; sarah.martin@qmul.ac.uk; 3 Department of Medical Biotechnologies, University of Siena, 53100 Siena, Italy; marcella.barbarino@unisi.it (M.B.); antonio.giordano@unisi.it (A.G.); 4 Translational Oncology, Center for Biotechnology, College of Science and Technology, Temple University, Sbarro Institute for Cancer Research and Molecular Medicine, Philadelphia, PA 19122, USA; luciano.mutti@temple.edu; 5 Department of Oncology, Transplantations and New Technologies in Medicine, University of Pisa, 56126 Pisa, Italy; andrea.nicolini@med.unipi.it; 6 Department of Medicine, Surgery and Neurosciences, Siena University Hospital, 53100 Siena, Italy; luca.luzzi@unisi.it; 7 Toma Institute Srl, Via Cesare Rosaroll 24, 80139 Napoli, Italy; lia@aiellogenetica.it

**Keywords:** malignant pleural mesothelioma, thonzonium bromide, cephalomannine, alexidine, ouabain, emetine, drug repositioning

## Abstract

**Simple Summary:**

Malignant pleural mesothelioma (MPM) is a disease of the pleura related to asbestos exposure. Despite the advancements in new therapeutic frontiers, it has a dismal prognosis and very limited treatment options. To find novel weapons in the care of MPM, we undertook a drug-repurposing approach that consists of evaluating existing drugs already approved for other human diseases. We screened 1170 drugs, and we observed that cephalomannine, a taxane; ouabain, a cardiac glycoside; thonzonium bromide, an antifungal surfactant; and emetine, an emetic alkaloid, had marked activity against immortalized and patient-derived primary MPM cell lines. These compounds were shown to be promising, and they will be evaluated in further studies, both in vitro and in vivo. We believe that drug repurposing is a valuable strategy to facilitate and accelerate the definition of novel treatment options for the management of MPM.

**Abstract:**

The lack of effective therapies remains one of the main challenges for malignant pleural mesothelioma (MPM). In this perspective, drug repositioning could accelerate the identification of novel treatments. We screened 1170 FDA-approved drugs on a SV40-immortalized mesothelial (MeT-5A) and five MPM (Mero-14, Mero-25, IST-Mes2, NCI-H28 and MSTO-211H) cell lines. Biological assays were carried out for 41 drugs, showing the highest cytotoxicity and for whom there were a complete lack of published literature in MPM. Cytotoxicity and caspase activation were evaluated with commercially available kits and cell proliferation was assayed using MTT assay and by clonogenic activity with standard protocols. Moreover, the five most effective drugs were further evaluated on patient-derived primary MPM cell lines. The most active molecules were cephalomannine, ouabain, alexidine, thonzonium bromide, and emetine. Except for alexidine, these drugs inhibited the clonogenic ability and caspase activation in all cancer lines tested. The proliferation was inhibited also on an extended panel of cell lines, including primary MPM cells. Thus, we suggest that cephalomannine, ouabain, thonzonium bromide, and emetine could represent novel candidates to be repurposed for improving the arsenal of therapeutic weapons in the fight against MPM.

## 1. Introduction

Malignant pleural mesothelioma (MPM) is a rare tumour of the pleura mainly caused by a past exposure to asbestos and often diagnosed in advanced stages [1]. As first-line chemotherapy, the European Society for Medical Oncology advised a regimen of pemetrexed and platinum (cisplatin/carboplatin) with folic acid and vitamin B12 supplementation [2,3]. However, despite some success with the use of novel agents, such as bevacizumab [4] or nintedanib [5,6] in a combinatory therapy, or with inhibitors of immune checkpoint molecules, most MPM patients still show an overall survival of about one year after the diagnosis [7,8,9,10,11,12]. For this reason, the so-called “drug repositioning” approach has become an additional appealing strategy to be exploited in this therapeutic context. In 2020, two repositionable drugs, quinacrine and metformin, have been proposed for the treatment of MPM patients. Quinacrine is an anti-malarial drug, and it induced apoptosis and inhibited cell migration, colony formation, and the autophagy process in immortalized and patient-derived primary MPM cell lines [13]. Metformin, a small molecule employed as first-line treatment in type-2 diabetes, when encapsulated in chitosomes as delivery carriers, caused an in vitro reduction of MPM cell viability and 3D-spheroid formation [14]. In line with this field of research, in a recent study we screened a library of 1170 FDA-approved drugs. This library of molecules was tested for their cytotoxic activity on a non-malignant SV40-immortalized mesothelial (MeT-5A) cell line and on five MPM cell lines. Our first strategy was aimed at selecting the set of molecules showing the highest activity on MPM cells, sparing the MeT-5A cell line. Thus, we found fludarabine and risedronic acid to be the most interesting molecules, as already reported [15]. However, we are aware that MeT-5A cells could not be the most appropriate model of non-malignant mesothelial cells. In fact, they show a high rate of proliferation and since their establishment in 1989, have developed changes for adaptation to in vitro culturing conditions. Moreover, one cell line alone could not be fully representative of the benign condition. In other words, a positive cytotoxic response elicited by the MeT-5A cell line should not prevent further investigation of additional active molecules on MPM cell lines. As a matter of fact, the drugs here within considered were already approved for use in humans, having a known, limited, and manageable toxicity profile. Therefore, in the present study we re-evaluated the results of the 1170 screened drugs and focused our attention on the set of molecules showing cytotoxic activity against MPM cell lines, irrespective of the toxicity measured on MeT-5A cells.

## 2. Materials and Methods

### 2.1. Mesothelioma Cell Lines

All the materials and methods have been published in detail on a previous manuscript [15]. Here, we report only a brief description, highlighting the main differences. The malignant cell lines employed were Mero-14, Mero-25, IST-Mes2, NCI-H28, MSTO-211H, Mero-41, Mero-95, ZL55, and REN. The non-malignant cell lines were MeT-5A and LP-9. The specific information on these cells can be found in the previous work [15]. However, here we extended the assays also on Mero-95, ZL55, and MMP4. Mero-95 and ZL55 were purchased from ECACC and cultured according to the manufacturer’s instructions. MMP4 mesothelioma cell lines were isolated from patients who underwent surgery at the Thoracic Surgery Unit (Siena, Italy) as in [16]. MMP4 cell lines were cultured at 37 °C and 5% CO_2_ in Medium 199 (Euroclone S.p.A., Milan, Italy), supplemented with 2 mmol/L l-glutamine (Euroclone S.p.A.), 20 ng/mL hEGF (Sigma-Aldrich, St. Louis, MO, USA), 100 U/mL penicillin, 10% FBS (Euroclone S.p.A.), 100 μg/mL streptomycin (Euroclone S.p.A.), and 0.4 μg/mL hydrocortisone (Sigma-Aldrich). The local Ethic Committee approved the study (Comitato Etico Regione Toscana-Area Vasta Sud Est; #CCMESOLUNG).

### 2.2. Compound Library Screen and Cell-Titer Assay

The FDA-approved drug library was composed of 1170 entities (Selleck Chemicals, Houston, TX, USA) [15]. Cell viability was assessed through the use of CellTiter-Glo^®^ Luminescent Cell Viability Assay (Promega, Madison, WI, USA). For the preliminary screening, all compounds are tested initially at a single dose of 10 μM, and only at a later stage, a selection of them will progress to the full five-dose assay. This one-dose screen methodological approach has already been used successfully before [15,17,18,19]. Drug screening was performed in duplicate. Stock solutions of the active drugs were prepared in DMSO, PBS, or sterile water and further validated by treating the cells with a range of concentrations (0.1 μM, 1 μM, 10 μM, and 100 μM). Then, the viability was measured with CellTiter-Glo^®^ Luminescent Cell Viability Assay, as reported previously [15]. Three independent experiments were carried out with three technical replicates.

### 2.3. Chemicals

Individual chemicals, i.e., albendazole, alexidine, azelastine, bosutinib, carmofur, cladribine, digoxigenin, dronedarone, emetine, miconazole, pentamidine, pitavastatin, sertraline, solifenacin, sulcanozole, tadalafil, terfenadine, thioridazine, trifluorothymidine, and toremifene were purchased from Cayman Chemical Company (Ann Arbor, MI, USA). Additionally, 10-deacetylbaccatin, allyl-thiourea, aminoacridine, bazedoxifene, cephalomannine, cetrimonium bromide, ciclopirox, clomifene, cytarabine, fenbendazole, ouabain, oxethazaine, penfluridol, pyrithione zinc, tioconazole, thioguanine, and vinorelbine were purchased from Sigma-Aldrich. Flubendazole was purchased from LKT Laboratories, Inc. (St. Paul, MN, USA), and gemcitabine and niclosamide were purchased from AdipoGen^®^ Life Science (Liestal, Switzerland), whereas thonzonium bromide and pralatrexate were purchased from MedChemExpress (Monmouth Junction, NJ, USA).

### 2.4. MTT (3-(4,5-Dimethylthiazolyl-2)-2,5-Diphenyltetrazolium Bromide), Sulphorodamine B (SRB), Colony Formation (CFA), and Caspase 3/7 Activation Assays

Drugs showing a dose-response activity on the previous assay were further studied for proliferation with MTT or SRB assays, both described in detail in the previous manuscript [15]. For CFA, the cells were treated, every three days, with 1 or 0.1 μM of the selected compounds by refreshing the medium and staining after ten days with SRB. The Caspase-Glo^®^ 3/7 Assay (Promega) was used for detecting the activation of caspases 3/7, a marker of apoptosis. The assay was carried out according to the manufacturer’s instruction and as reported in the previous study [15]. Luminescence was detected with FLUOstar^®^ Omega microplate reader (BMG LabTech, Offenburg, Germany). Three independent experiments with three technical replicates each were carried out.

### 2.5. Spheroid Formation Assay

Among our panel of cells, we firstly assessed that only MeT-5A, Mero-25, IST-Mes2, MSTO-211H, HMC7, MMP1, and MMP4 were able to form spheroids. Cells were seeded in Nunclon™ Sphera™ 96-Well microplates (Thermo Fisher Scientific, Waltham, MA, USA), allowing spheroids to aggregate for 48 h before treatments with either controls or drugs. TB and CM were used at concentrations of 0.2, 1, and 5 μM, whereas OB and EM at concentrations of 0.025, 0.05, and 0.1 μM. All treatments lasted for 72 h. Spheroids were kept at 37 °C and 5% CO_2_, and growth was monitored daily and recorded using a light microscope (ZEISS, Jena, Germany). Then, after excluding aggregates of outliers showing extra-large dimensions or unusual shapes, frankly isolated spheroids were analysed at the microscope for their size or morphology. The size was measured as area occupied on the surface of the visual field by counting (with the software ImageJ) the total number of pixels covered by spheroids. Then, the average spheroid area in each well was calculated as a ratio of the total pixels shared by the total number of spheroids (counted manually) and expressed as “spheroid average size” (arbitrary unit). Alternatively, we used the grade of damage to spheroids, seen as sloughing of outer layers. The average damage in each well was measured as the ratio of the “number of damaged spheroids/total number of spheroids” and expressed as “morphological score” (arbitrary unit). Both methods of counting were carried out by two independent readers blinded for the state of spheroids. Data were reported as mean ± standard error of the mean (SEM) of two experiments, each performed in duplicate.

### 2.6. Statistical Analysis

Similar to what was reported in [15], the values of the measured luminescence were log-transformed and normalized using the median signal as the reference and standardized with a Z-score statistic. For this normalization procedure, the median absolute deviation was employed to determine the variation in each screen. A Z-score value < −1.96 in at least four out of five MPM cell lines was used as the criteria to consider a compound cytotoxic in a specific cell line. The difference of viability between controls and treated cultures at various doses was carried out with the Dunnett’s multiple comparisons test. The calculation of the IC_50_s was carried out with the software GraphPad Prism 7 (San Diego, CA, USA). To rank drugs according to the overall activity, the −Log_10_ of the IC_50_s summed across all the cell lines was calculated.

For cell proliferation, the ODs obtained from three independent experiments (each using three technical replicates) were analysed with the multivariate analysis of variance (MANOVA) with interaction (using treatment and experimental times as the independent factors). Within this type of analysis, the differences between control and treatments at each dose were obtained through the Sidak multiple comparison test. CFA, caspase activation, and spheroids’ formation assays were evaluated with the one-way analysis of variance and the Dunnett’s multiple comparisons test. The statistical analyses were carried out with GraphPad Prism 7 and with Statgraphics Centurion XVI (version 16.1.11; Statgraphics Technologies, Inc. The Plains, VA, USA). A *p*-value of 0.05 was employed as the statistical threshold of significance.

## 3. Results

### 3.1. Preliminary Cytotoxicity Screening

In the present study we re-evaluated the results of our recent screening of 1170 FDA-approved drugs [15]. It was carried out by measuring the cytotoxicity at the single dose of 10 μM for 72 h using the CellTiter Glo^®^ assay, and it was performed on MeT-5A (a known model of non-malignant cell line from pleura) and five permanent MPM cell lines. The re-evaluation allowed us to identify 90 cytotoxic drugs active in at least four out of five MPM cell lines and to a various extent, also in the MeT-5A cell line. The results of these 90 molecules are reported in Table S1.

### 3.2. Dose-Response Cytotoxicity Assays

According to the published literature, 56 of the 90 drugs were never assayed in MPM models (either in vitro or in vivo). Thus, we selected 38 of these drugs and evaluated whether their cytotoxicity could be validated in a dose-dependent manner (using 0.1, 1, 10, and 100 μM) in the MeT-5A, Mero-14, Mero-25, IST-Mes2, NCI-H28, and MSTO-211H cell lines. As a control, we also employed four of the thirty-four positive molecules previously published as being cytotoxic in MPM cells: cytarabine, gemcitabine, pralatrexate, and vinorelbine.

The complete set of dose–response curves used for the calculation of the IC_50_s are reported in Supplementary Materials (“Supplementary dose-response”). To provide a unique measure of the cytotoxic activity for each drug, the IC_50_s were added together from across all cell lines and their −Log_10_ were calculated. Thus, the overall activity of these compounds and their rankings are represented in Figure S1, while the 20 most active molecules are highlighted in Table 1.

**Figure 1:**
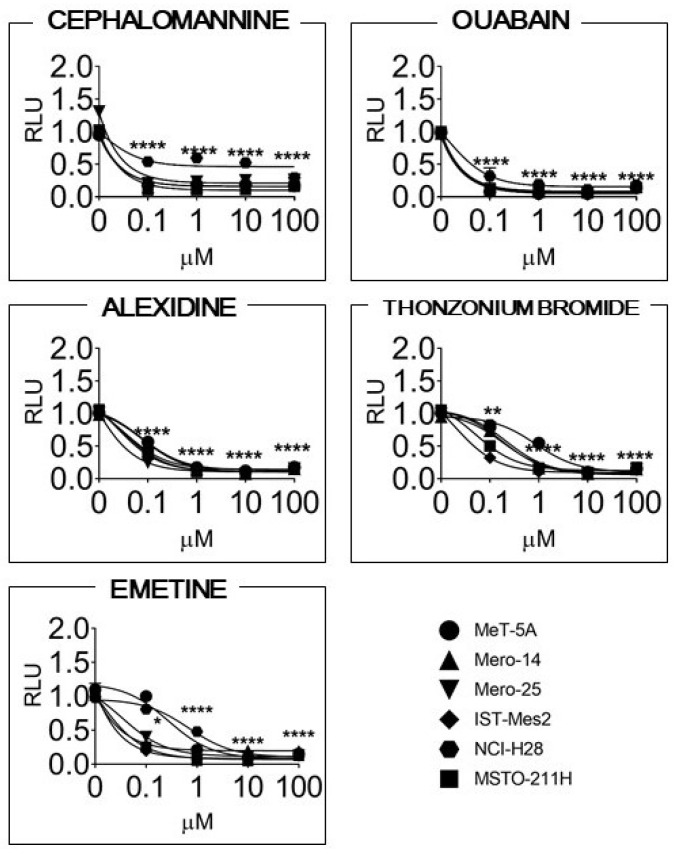
Cell viability, expressed as relative luminescence units (RLU) (i.e., the luminescence relative to the controls) and measured at 72 h after treatment of the cells with the doses of 0, 0.1, 1, 10, and 100 μM of each compound. Three independent experiments, each performed in triplicate, were carried out. Dots represent the means, their size, and the SEM. Asterisks (*) represent the statistical significance of the comparison between the considered dose and the control, where * = *p* < 0.05; ** = *p* < 0.01, and **** = *p* < 0.0001.

Among the compounds cytotoxic at the lowest doses, there were several molecules known to be active on MPM, such as pralatrexate, gemcitabine, cytarabine, and vinorelbine. However, in the present work, we deepen the investigation on cephalomannine (CM; a taxane), ouabain (OB; a cardiac glucoside), alexidine (AX; an antimicrobial), thonzonium bromide (TB; an antifungal surfactant and inhibitor of the vacuolar ATPases), and emetine (EM; anthelmintic), as they showed the strongest cytotoxicity compared to the other drugs. In any case, the remaining molecules are still of interest, and they could be the objects of future studies. The dose–response curves for CM, OB, AX, TB, and EM are shown in Figure 1 and were used to determine the lowest, most effective dose common to all cell lines (i.e., 0.1μM for CM, EM, and OB and 1μM for TB and AX) to be employed in further assays.

### 3.3. Proliferation, Caspase Activation, and Colony Formation Assays

**Figure 2:**
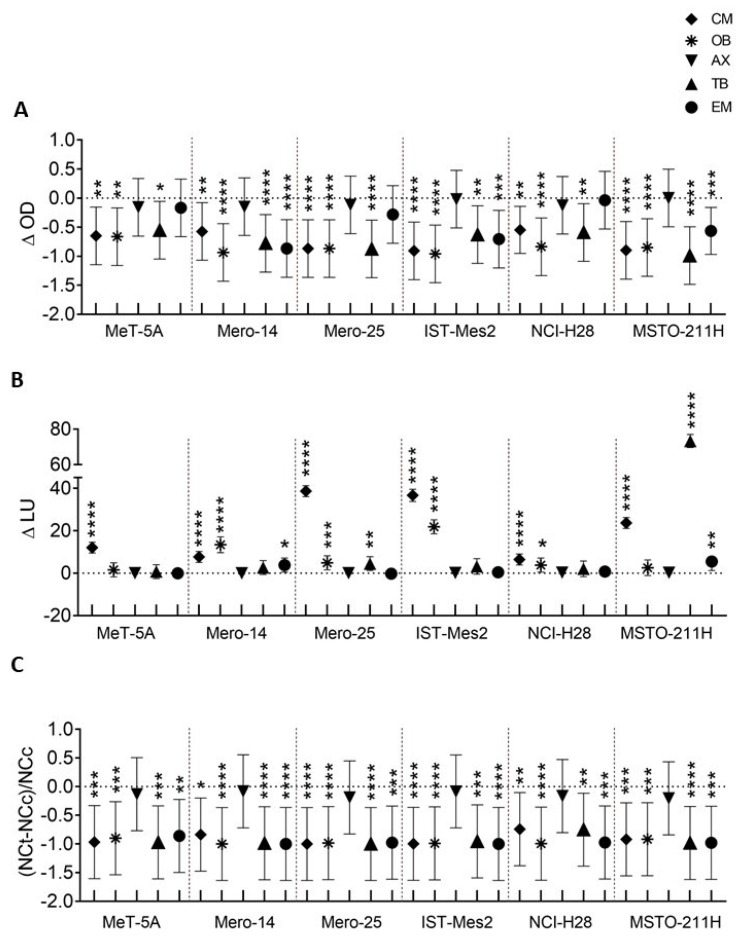
In vitro evaluation of CM (at 0.1 μM), OB (at 0.1 μM), AX (at 0.1 μM), TB (at 1 μM), and EM (at 0.1 μM) in MeT-5A mesothelial cell line and in Mero-14, Mero-25, IST-Mes2, NCI-H28, and MSTO-211H MPM cell lines (depicted in the *X*-axis). (**A**) *MTT assay for cell viability*. *Y*-axis reports the average difference as dots (and its confidence interval as whiskers) between the optical densities (OD) measured after 72 h in treated and control cultures. The extended timeline graph is represented in Figures S2 (CM), S4 (OB), S6 (AX), S8 (TB), and S10 (EM). (**B**) *Caspases’ activation assay for apoptosis*. *Y*-axis reports the average difference (∆LU as dots) and its confidence interval (as whiskers) between the units of luminescence obtained with the Caspase-Glo^®^ 3/7 assay and measured after 72 h in treated and control cultures. The extended timeline graph, representing the activation of caspases after 24, 48, and 72 h of treatment, is shown in Figures S3a (CM), S5a (OB), S7a (AX), S9a (TB), and S11a (EM). (**C**) *CFA for clonogenic activity*. The *Y*-axis reports the relative difference in the number of colonies counted in the treated (NCt) and control (NCc) cultures and expressed as (NCt-NCc)/NCc. Colonies were counted 11 days after the treatment. Counts were carried out with ImageJ digital processing software and a representative picture (for visual purposes only) can be found in Figures S3b (CM), S5b (OB), S7b (AX), S9b (TB), and S11b (EM). In (**A**–**C**), data are reported as the mean and its confidence intervals of three independent experiments. All datapoints with the confidence interval not encompassing the dotted line are statistically significant at various extents where * = *p* < 0.05; ** = *p* < 0.01; *** = *p* < 0.001, and **** = *p* < 0.0001.

We further analyzed CM, OB, AX, TB, and EM for their ability to inhibit the proliferation and the clonogenicity in our cell lines and their ability to induce the activation of caspases after treatment. **(a) CM**. CM caused decreased proliferation, statistically significant in all cell lines at 72 h after the treatment (Figure 2A and Figure S2). Furthermore, at 72 h after treatment, CM caused a statistically significant activation of caspases-3 and -7 in all cell lines, with the strongest response observed in Mero-25 and IST-Mes2 (*p* < 0.0001) (Figure 2B and Figure S3a). However, for all cells analyzed, the results were already statistically significant at 48 h (Figure S3a). Finally, treatment with CM inhibited the colony formation ability in all cell lines (*p* < 0.0001) (Figure 2C and Figure S3b). **(b) OB**. A statistically significant decrease of proliferation was observed in all cell lines analyzed, starting from 48 h after the treatment (Figure 2A and Figure S4). OB also caused a highly significant activation of caspases, except in MeT-5A and MSTO-211H (Figure 2B and Figure S5a). Figure 2C and Figure S5b show the significant reduction in the number of colonies formed in cells treated with OB at a concentration of 0.1 μM, as compared to the respective control (*p* < 0.0001). **(c) AX**. Treatment with AX at 0.1 μM did not elicit significant changes in any cell line under investigation, neither in the proliferation (Figure 2A and Figure S6) nor in the caspase activation (Figure 2B and Figure S7a) nor in the clonogenic assay (Figure 2C and Figure S7b). For this reason, we decided not to continue further with the analysis of AX. **(d) TB**. Upon treatment of 1 μM TB, we observed an inhibition of the growth of MPM cell lines following 72 h of culturing post treatment (Figure 2A and Figure S8). MeT-5A cells displayed a capacity to grow after TB treatment, although to a lesser extent than when treated with the vehicle alone (Figure S8). This different behavior was highlighted by a high statistical significance of the comparison between treated cells and controls in MPM cells, while the statistical significance of MeT-5A was barely close to 0.05 (Figure S8). In addition, in Mero-25 and MSTO-211H cell lines, TB caused a statistically significant activation of caspase-3 and -7 at 72 h after the treatment (*p* < 0.0001) (Figure 2B and Figure S9a). Finally, the treatment with TB inhibited colony formation in all cell lines (*p* < 0.0001) (Figure 2C and Figure S9b). **(e) EM**. Treatment with EM caused a statistically significant reduction of the proliferation of Mero-14, IST-Mes2, and MSTO-211H at 72 h (*p* < 0.0001) (Figure 2A and Figure S10). EM also induced a weak activation of caspases-3 and -7, statistically significant in Mero-14 and MSTO-211H (Figure 2B and Figure S11a; *p* < 0.01). In addition, EM prevented all the cell lines from forming colonies (*p* < 0.0001) (Figure 2C and Figure S11b).

### 3.4. Evaluation of CM, EM, OB, and TB on a Panel of Epithelioid MPM Cells

The toxicity of the selected molecules was further assayed by using the same doses on a different panel of MPM cell lines of epithelioid origin (Mero-41, Mero-95, ZL55, and REN), two non-malignant cell lines (i.e., LP-9, immortalized and commercially available, and HMC7, a primary cell line derived from a spontaneous pneumothorax), and three primary cell lines derived from MPM patients (i.e., MMP1, MMP2, and MMP4).

**Figure 3:**
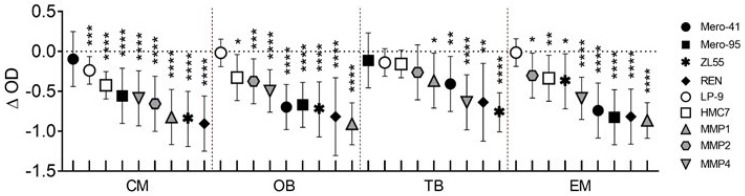
Evaluation of CM, OB, TB, and EM with MTT assay for cell viability carried out on a second set of cell lines. *Y*-axis reports the average difference (as dots) and its confidence interval (as whiskers) between the optical densities (OD) measured after 72 h in treated and control cultures. Drugs were assayed at the same doses employed in the first set of cell lines (i.e., CM, 0.1 μM; OB, 0.1 μM; TB, 1 μM; EM, 0.1 μM) and the correspondences are: (i) black dots, permanent MPM cell lines, (ii) white dots, cultures of non-malignant pleural cells, and (iii) grey dots, primary MPM cell lines. Data are reported as mean and its confidence intervals of three independent experiments. When the differences were statistically significant, their divergence from 0 were represented by asterisks, as * = *p* < 0.05; ** = *p* < 0.01; *** = *p* < 0.001, and **** = *p* < 0.0001.

After 72 h of treatment, a statistically significant reduction in cell viability caused by the drugs analysed was confirmed in almost all the MPM cell lines, except for CM in Mero-41 and TB in Mero-95 (Figure 3, black dots). The viability of the non-malignant cells was affected to a much lesser extent, as displayed by the ∆OD values in Figure 3 for the white dots. In particular, we observed a statistically significant reduced viability only for HMC7 treated with CM and EM, while LP-9 responded only to CM treatment. Finally, the patient-derived MPM cell cultures responded to all drugs, except MMP2 treated with TB. Overall, the reduced viability was similar to that measured in immortalized MPM cell lines, as reported by the grey dots of Figure 3.

### 3.5. Effect of CM, EM, OB, and TB on 3D Spheroids Derived from MPM Cells

We furthered the investigation of these molecules by performing a spheroid formation assay. Spheroids are considered models of 3D structures that could approximate the cellular organization within a solid mass, and it could provide additional information on the activities of these molecules. Spheroids originated from MeT-5A, Mero-25, IST-Mes2, and MSTO-211H cell lines were established and treated with increasing doses of CM, CB, TB, and EM. The 3D spheroids displayed altered morphology and reduced circularity and compactness, particularly on the borders, as shown by the increased morphological score (Figure S12) and by the representative images (Figure S13). Moreover, spheroids originated from HMC7, MMP1, and MMP4 cell lines showed a dose-dependent reduced average size, except HMC7 treated with OB or EM, and MMP4 treated with OB (see Figure S14). In Figure S15 representative pictures of these cells are reported.

## 4. Discussion

The goal of the present study was the identification of novel drugs active against the malignant behavior of MPM cells with a drug-repurposing approach. Thus, 1170 FDA-approved drugs were screened on one non-MPM (MeT-5A) and five MPM (Mero-14, Mero-25, IST-Mes2, MSTO-211H, NCI-H28) cell lines, and CM, OB, AX, TB, and EM were among the most active. The activity was further confirmed for all the molecules, except for AX, in other in vitro systems. We are aware that the preliminary screening performed at a single dose of 10 μM could poses a bias in the selection of the best active molecules. In fact, some drugs could be effective against MPM at higher doses or eliciting mechanisms not detected through the set of assays employed in this paper (e.g., by blocking the cell cycle progression). Moreover, since a single-dose assay could be less informative about the toxic activity of a drug than a full-range dose assay, some promising candidates may have been missed. However, the FDA-approved drug library employed in this work has been successfully used at this concentration previously [18,19], and has led to its translation into a clinical trial [20]. Another limitation of the present study could be the reduced availability of primary non-malignant cells derived from normal pleura. We surrogated these controls by using immortalized non-malignant cell lines or cultures derived from biopsies of non-MPM patients, but it should be warned that this type of control could bias the results. Despite these limitations, we identified molecules with promising activities on MPM and are worth further investigations, also with in vivo models. In particular, novel studies are needed to define the therapeutic window where compounds could be most effective with the least in vivo toxicity. CM is a taxane isolated from the needles of *Taxus canadensis* [21,22,23]. The activity of taxanes as anticancer agents has a long history and is well documented. These agents act as microtubule stabilizers and disrupt microtubule dynamics, thus inducing mitotic arrest and ultimately, cell death by apoptosis [24,25]. Its naturally occurring analogue paclitaxel (that differs only in its C-13 side chain) has been found active on MPM patients. Therefore, we also showed that this taxane could be an alternative to paclitaxel in the therapeutic care of MPM [26]. TB is a surfactant agent, and it is employed as a preservative for its anti-microbial activity. TB was shown to act against methicillin-resistant *Staphylococcus aureus* strains [27], *Cooperia oncophora* (a helminth) [28], and *Candida albicans* where it can inhibit the vacuolar “V” ATPase [29,30]. Here we suggest for the first time, that TB could be active against MPM cell lines. No one before described this molecule as an anticancer agent; as a matter of fact, there is very limited knowledge on its possible activities on mammalian cells. A study by Zhu X. et al. in 2016 [31] showed, both in vitro and in vivo, that TB has anti-osteoclastogenic activity by blocking the RANKL-induced activation of the MAPK-signaling pathways (ERK, JNK and p38). In the present work, TB appeared the least active on non-malignant cell lines MeT-5A, LP-9, and HMC7, showing the highest differential toxicity towards the malignant cells. It could be speculated that this toxicity is related to the deregulation of the above-mentioned pathways in MPM [32,33]. EM is an anti-protozoal alkaloid [34] extracted from the roots of the *Psychotria ipecacuanha* plant. It disturbs DNA, RNA, and protein synthesis [35,36,37], and it was shown to induce apoptosis in in vitro cultures of cancer cells [38]. OB is a cardiac glycoside from the seed of the *Strophantus gratus* plant. Based on its positive ionotropic activity occurring via the inhibition of Na/K-ATPase, OB is used in the treatment of congestive heart failure and cardiac arrhythmias [39,40]. However, it also showed promise as an anticancer agent when tested on leukaemia, breast cancer, and non-small cell lung cancer [41].

Drug repurposing could represent a valuable strategy, especially when alternatives are not available for the cure of unmet diseases, such as MPM. Theoretically, CM, OB, and TB should be further investigated with in vivo studies followed by clinical trials. Despite the reduced costs, the clinical research of repurposed drugs could still be challenging, as further studies need to be carried out on formulations, doses, posology, and toxicity profiles. For example, TB has been approved for topic uses, but its effects when administered through other modalities is not known. Moreover, it would be very important to add knowledge on the interactions with other chemotherapeutic agents or in the context of immuno-therapy, a type of research that is very time demanding and resource consuming. Since immuno-therapy was shown to be less effective in MPM than in other tumors [42], novel combinations of drugs are needed for obtaining crucial improvements. In this context, any novel effective compound represents a promising candidate to be added to standard chemotherapy or to innovative immuno-therapies for widening the set of treatment options in the fight against MPM. Especially in the context of rare diseases, such as MPM, we should further consider that the patent expiry for these drugs could discourage appropriate investments for their repositioning, thus hampering a translation into clinical trials. In any case, we still aim to evaluate the potentially repositionable compounds also with in vivo models. This could provide further evidence on the potential benefits of known drugs in the treatment of MPM and encourage further studies from independent parties.

## 5. Conclusions

In conclusion, the present study allowed the identification of several molecules active against MPM cell lines never previously reported with such an activity. However, it should not be forgotten that there were more molecules placed lower in the rank that appeared potentially interesting and worthy of an evaluation in future experiments. Here, CM, OB, TB, and EM showed marked activity on MPM cells, warranting their evaluation in future in vitro and in vivo experiments, also to ascertain their underlying mechanisms of action. Once additional evidence on their effectiveness is proven, it could be foreseen as an application in a potential combined therapy.

## Figures and Tables

**Table 1:** Overall activity for the 20 most cytotoxic drugs. The first column lists the selected 20 most cytotoxic compounds among the 90 assayed. The table reports the IC_50_ (μM) measured on each cell line. The drugs were ranked according to the overall activity calculated as the −Log_10_ of the sum of the IC_50_s.

	MeT-5A	Mero-14	Mero-25	IST-Mes2	NCI-H28	MSTO-211H	−Log_10_(∑(IC_50_))
Cephalomannine	0.01	0.01	0.01	0.01	0.04	0.01	1.05
Pralatrexate	0.02	0.01	0.01	0.02	0.07	0.01	0.85
Ouabain	0.05	0.02	0.01	0.01	0.01	0.05	0.82
Alexidine HCl	0.09	0.09	0.07	0.06	0.04	0.08	0.37
Gemcitabine	0.00	0.14	0.01	0.04	0.23	0.01	0.37
Cytarabine	0.09	0.38	0.12	0.03	0.07	0.05	0.13
Vinorelbine	0.06	0.01	0.01	0.01	0.98	0.01	−0.03
Thonzonium Br	0.74	0.19	0.17	0.05	0.21	0.07	−0.16
Emetine	0.73	0.07	0.03	0.06	0.63	0.08	−0.18
Pyrithione zinc	0.27	0.67	0.90	0.07	0.71	0.37	−0.48
Cetrimonium Br	0.90	0.88	0.33	0.39	1.01	0.01	−0.55
Fenbendazole	0.46	0.60	0.27	0.86	1.22	0.17	−0.55
Pentamidine	1.54	0.47	1.25	0.52	0.18	0.10	−0.61
Niclosamide	0.82	0.69	0.78	0.86	0.76	0.40	−0.63
Pitavastatin Ca	0.11	0.07	3.22	0.90	0.25	0.11	−0.67
Ciclopirox	0.12	0.91	0.91	1.00	1.71	0.22	−0.69
Terfenadine	1.35	0.53	1.24	1.06	1.43	0.16	−0.76
Penfluridol	1.27	1.08	1.04	0.64	1.33	0.44	−0.76
Albendazole	0.88	0.48	0.44	0.41	5.22	0.11	−0.88
Digoxigenin	0.19	0.71	0.97	0.24	2.70	5.66	−1.02

## Data Availability

The data that support the findings of this study are available from the corresponding author upon reasonable request.

## Supplementary Materials

The following are available online at https://www.mdpi.com/article/10.3390/cancers14102527/s1, “Supplementary_dose-respoonse” reports the complete set of dose–response curves used for the calculation of the IC_50_s. Table S1: Z-scores of 90 compounds listed in alphabetical order showing cytotoxic activity in at least four MPM cell lines. The table also reports the response of the non-malignant MeT-5A cell line; Figure S1: IC_50_-based ranking of drugs assessed by dose–response cytotoxicity assays; Figure S2: MTT Assay for CM; Figure S3: Caspases and CFA assays after treatment with CM at 0.1 μM in MeT-5A mesothelial cell line and in Mero-14, Mero-25, IST-Mes2, NCI-H28, and MSTO-211H MPM cell lines; Figure S4: MTT Assay for OB; Figure S5: Caspases and CFA assays after treatment with OB at 0.1 μM in MeT-5A mesothelial cell line and in Mero-14, Mero-25, IST-Mes2, NCI-H28, and MSTO-211H MPM cell lines;. Figure S6: MTT Assay and AX; Figure S7: Caspases and CFA assays after treatment with AX at 0.1 μM in MeT-5A mesothelial cell line and in Mero-14, Mero-25, IST-Mes2, NCI-H28, and MSTO-211H MPM cell lines; Figure S8: MTT Assay for TB; Figure S9: Caspases and CFA assays after treatment with TB at 1 μM in MeT-5A mesothelial cell line and in Mero-14, Mero-25, IST-Mes2, NCI-H28, and MSTO-211H MPM cell lines; Figure S10: MTT Assay for EM; Figure S11: Caspases and CFA assays after treatment with EM at 0.1 μM in MeT-5A mesothelial cell line and in Mero-14, Mero-25, IST-Mes2, NCI-H28, and MSTO-211H MPM cell lines; Figure S12: Spheroid assay on MeT-5A mesothelial cell line and Mero-25, IST-Mes2, and MSTO-211H MPM cell lines; Figure S13: Spheroid assay on MeT-5A and Mero-25, IST-Mes2, and MSTO-211H MPM cells; Figure S14: Spheroid assay on patient non-immortalized HMC7 and tumor MMP1 and MMP4 cells; Figure S15: Spheroid assay on patient non-immortalized HMC7 and tumor MMP1 and MMP4 cells.
