# Supplementary material for: A Drug Screening Revealed Novel Potential Agents against Malignant Pleural Mesothelioma
Source: Cancers (Basel). 2022 May 20;14(10):2527. doi: 10.3390/cancers14102527 (PMC9139775; doi:10.3390/cancers14102527)
Supplement: Supplementary file 1 [file cancers-14-02527-s001.zip › cancers-1704721-supplementary.pdf]

Table S1: Z-scores of 90 compounds, listed in alphabetical order, showing cytotoxic activity in at least four MPM cell lines. The table also reports the response of the non-malignant MeT-5A cell line. The mean and the standard error of the mean (SEM) derive from two independent experiments.

	**MeT-5A**		**Mero-14**		**Mero-25**		**IST-MES2**		**NCI-H28**		**MSTO-211H**	
	Mean	SEM	Mean	SEM	Mean	SEM	Mean	SEM	Mean	SEM	Mean	SEM
**10-Deacetylbaccatin**	-6,78	±0,45	-3,46	±0,70	-5,34	±0,04	-6,16	±0,17	-3,19	±0,16	-2,21	±0,47
**Aminoacridine**	-5,10	±0,38	-3,60	±0,18	-3,58	±0,01	-4,12	±0,00	-6,27	±0,02	-3,08	±1,15
**Afatinib**	-5,06	±3,95	-7,24	±0,40	-8,12	±0,63	-8,02	±0,05	-11,16	±1,03	-6,37	±1,12
**Albendazole**	-5,99	±0,17	-4,01	±0,33	-5,85	±0,05	-6,15	±0,14	-6,56	±0,37	-1,62	±1,06
**Alexidine HCl**	-6,30	±0,26	-4,46	±0,27	-4,97	±0,04	-5,10	±0,06	-7,38	±0,03	-1,92	±0,71
**Alfuzosin HCl**	-3,67	±0,09	-2,28	±0,40	-2,65	±0,77	-3,25	±0,76	-3,12	±0,24	-2,07	±0,80
**Allylthiourea**	-11,59	±3,28	-10,25	±0,32	-10,70	±0,73	-11,47	±0,83	-14,51	±3,02	-3,77	±1,89
**Auranofin**	-5,51	±0,47	-3,75	±0,37	-4,01	±0,19	-3,85	±0,02	-6,12	±0,18	-3,22	±1,08
**Azelastine HCl**	-3,09	±0,19	-6,16	±0,89	-5,11	±0,75	-5,68	±1,44	-4,56	±0,15	-4,71	±0,88
**Bazedoxifene HCl**	-4,28	±0,17	-3,69	±0,46	-3,94	±0,16	-3,93	±0,18	-2,67	±2,88	-2,42	±0,94
**Benzethonium HCl**	-5,26	±0,24	-3,72	±0,45	-3,80	±0,08	-3,60	±0,28	-3,01	±3,54	-2,30	±0,18
**Bortezomib**	-9,17	±0,70	-8,87	±0,64	-9,63	±0,79	-9,43	±0,86	-12,98	±1,51	-7,86	±2,12
**Bosutinib**	-6,06	±0,75	-7,88	±0,45	-8,49	±0,67	-7,49	±0,21	-10,80	±1,99	-7,37	±1,76
**Cabazitaxel**	-3,61	±0,30	-2,51	±0,22	-3,46	±0,01	-3,63	±0,29	-4,00	±0,08	-2,23	±0,69
**Camptothecin**	-5,56	±0,27	-5,04	±0,39	-4,63	±0,38	-4,80	±0,14	-7,24	±0,01	-2,98	±1,08
**Carfilzomib**	-6,83	±0,22	-4,97	±0,47	-5,50	±0,25	-6,08	±0,55	-7,46	±0,54	-4,06	±0,81
**Carmofur**	-2,09	±0,28	-2,89	±0,25	-2,58	±0,11	-3,97	±0,34	-3,25	±0,22	-2,54	±1,26
**Cephalomannine**	-10,39	±0,84	-5,87	±0,36	-8,79	±0,29	-9,91	±0,11	-9,04	±0,66	-5,49	±1,19
**Cepharanthine**	-2,25	±1,68	-4,04	±0,02	-2,93	±0,58	-3,95	±0,01	-3,33	±2,00	-3,62	±1,23
**Cetrimonium HBr**	-6,23	±0,04	-4,26	±0,18	-4,73	±0,21	-4,84	±0,13	-6,80	±0,29	-3,67	±0,83
**Cetylpyridinium HCl**	-5,00	±0,02	-4,08	±0,35	-4,04	±0,29	-4,41	±0,25	-5,62	±0,40	-0,96	±1,13
**Chlorquinaldol**	-2,84	±0,00	-2,84	±0,14	-3,27	±0,03	-3,94	±0,11	-2,58	±0,09	-3,10	±0,90
**Ciclopirox**	-3,35	±0,32	-3,40	±0,10	-3,47	±0,02	-3,96	±0,01	-4,39	±0,32	-2,59	±1,08
**Cladribine**	-3,01	±0,39	-2,97	±0,12	-2,58	±0,12	-3,40	±0,06	-2,60	±0,25	-1,30	±1,67
**Clofazimine**	-3,59	±0,40	-2,33	±0,48	-2,92	±0,43	-3,55	±0,67	-2,70	±0,02	-1,78	±1,68
**Clomifene citrate**	-3,81	±0,04	-6,04	±0,84	-6,10	±0,09	-6,11	±0,44	-3,07	±1,24	-0,77	±0,29
**Crizotinib**	-3,30	±0,05	-2,56	±0,16	-2,76	±0,64	-2,38	±0,03	-4,49	±0,26	-2,69	±0,37
**Crystal violet**	-7,88	±0,13	-5,74	±0,59	-6,40	±0,17	-6,48	±0,22	-9,83	±0,60	-4,62	±1,39
**Cyclamic acid**	-6,28	±3,62	-5,14	±2,96	-4,95	±3,73	-5,61	±4,17	-8,17	±5,89	-2,72	±1,40
**Cytarabine**	-3,59	±1,48	-3,46	±0,75	-4,05	±0,30	-4,47	±0,24	-3,30	±2,19	-1,68	±0,88
**Cytidine**	-3,52	±0,10	-2,16	±0,41	-2,19	±0,16	-2,67	±0,67	-3,56	±0,29	-1,92	±0,99
**Dasatinib**	-3,53	±0,17	-5,28	±0,70	-5,14	±0,12	-5,49	±0,88	-9,05	±0,63	-6,79	±2,27
**Daunorubicin HCl**	-6,12	±0,05	-4,62	±0,50	-4,96	±0,04	-5,15	±0,03	-6,62	±0,43	-4,03	±1,57
**Dequalinium HCl**	-4,61	±0,10	-3,10	±0,14	-3,23	±0,13	-3,63	±0,11	-3,08	±2,89	-2,64	±1,39
**Digoxigenin**	-4,95	±0,05	-4,81	±0,08	-4,86	±0,34	-4,59	±0,05	-6,03	±0,60	-3,39	±0,72
**Disulfiram**	-3,33	±0,83	-3,55	±0,61	-3,24	±0,29	-3,94	±0,04	-2,75	±1,27	-2,97	±0,76
**Docetaxel**	-3,38	±0,92	-1,98	±0,13	-2,30	±0,21	-3,30	±0,12	-3,55	±0,31	-1,53	±0,53
**Domiphen**	-5,17	±0,05	-3,62	±0,65	-3,52	±0,07	-3,48	±0,25	-5,92	±0,17	-1,07	±0,65
**Doxorubicin**	-4,29	±0,44	-3,26	±0,10	-2,72	±0,40	-3,92	±0,11	-5,44	±0,59	-1,69	±0,98
**Doxycycline HCl**	-4,90	±3,55	-5,52	±0,09	-5,76	±0,05	-7,44	±1,21	-5,98	±4,82	-1,19	±1,74
**Dronedarone HCl**	-5,09	±0,36	-3,94	±0,31	-4,00	±0,28	-4,15	±0,10	-3,55	±3,00	-2,99	±1,04
**Emetine**	-4,73	±0,16	-3,33	±0,04	-3,65	±0,16	-3,99	±0,07	-5,20	±0,40	-2,59	±1,02
**Epirubicin HCl**	-4,67	±0,53	-2,66	±0,11	-2,66	±0,40	-3,22	±0,19	-4,58	±0,16	-1,45	±0,58
**Fenbendazole**	-5,18	±0,17	-2,80	±0,46	-4,67	±0,06	-4,82	±0,00	-5,28	±0,86	-3,70	±0,44
**Fidaxomicin**	-3,27	±1,01	-3,90	±0,43	-2,00	±0,41	-4,54	±0,12	-2,57	±1,33	-2,20	±1,02
**Flubendazole**	-4,78	±0,65	-3,06	±0,41	-3,38	±0,52	-3,11	±1,73	-3,87	±0,70	-2,95	±1,46
**Fluvastatin Na**	-4,21	±0,19	-3,83	±0,87	-2,07	±0,80	-1,94	±0,96	-2,89	±1,34	-3,68	±0,95
**Gemcitabine HCl**	-4,39	±0,71	-2,41	±0,04	-2,37	±0,26	-3,42	±0,05	-4,92	±0,04	-1,33	±0,07
**Idarubicin HCl**	-4,84	±0,28	-3,62	±0,40	-4,37	±0,43	-4,83	±0,23	-6,55	±0,69	-3,27	±1,34
**Irinotecan HCl $3H_20$**	-4,95	±0,27	-3,03	±0,43	-3,55	±0,23	-3,93	±0,12	-2,64	±1,46	-2,49	±0,50
**Miconazole**	-4,88	±0,38	-3,62	±0,42	-3,72	±0,21	-3,68	±0,28	-5,58	±0,23	-0,18	±0,82
**Mitoxantrone HCl**	-5,39	±0,04	-4,10	±0,06	-4,15	±0,25	-3,70	±0,44	-5,76	±0,21	-3,12	±0,79
**Mycophenolate**	-3,56	±0,09	-2,39	±0,11	-2,55	±0,17	-2,94	±0,31	-3,28	±0,11	-2,53	±0,73
**Mycophenolic**	-2,58	±0,78	-2,90	±0,22	-3,07	±0,11	-3,89	±0,03	-4,09	±0,04	-3,71	±0,92
**Nebivolol**	-2,12	±2,76	-2,23	±1,19	-2,38	±2,49	-2,95	±1,83	-2,05	±1,90	-2,97	±0,71
**Niclosamide**	-4,70	±0,13	-3,48	±0,38	-3,79	±0,16	-3,82	±0,06	-5,80	±0,58	-2,89	±0,04

**Otilonium HBr**	-2,39	±0,03	-2,01	±0,57	-2,25	±0,46	-2,33	±0,05	-2,24	±0,16	-2,09	±0,40
**Ouabain**	-10,37	±2,77	-5,67	±3,42	-6,27	±4,05	-6,74	±4,07	-12,12	±3,20	-3,62	±1,55
**Oxethazaine**	-3,69	±1,80	-2,31	±0,93	-2,91	±1,02	-3,75	±0,41	-2,92	±1,14	-2,75	±0,40
**Oxibendazole**	-2,97	±0,10	-2,21	±0,36	-2,59	±0,76	-3,40	±0,04	-3,55	±0,32	-1,77	±0,55
**Palonosetron HCl**	-2,85	±0,06	-3,01	±0,87	-2,46	±0,09	-3,80	±0,19	-2,30	±0,20	-1,86	±1,12
**Penfluridol**	-6,54	±0,15	-4,51	±0,33	-4,86	±0,08	-4,70	±0,35	-6,76	±0,99	-3,57	±1,10
**Pentamidine**	-3,10	±0,08	-3,80	±1,77	-3,46	±1,35	-5,55	±0,33	-6,47	±3,98	-2,68	±0,68
**Pimobendan**	-2,69	±0,18	-2,36	±0,25	-2,84	±0,46	-3,26	±0,28	-1,49	±0,41	-2,64	±0,63
**Pitavastatin Ca**	-6,31	±0,30	-5,91	±0,76	-3,28	±0,78	-3,80	±0,84	-5,75	±0,06	-3,31	±0,05
**Pralatrexate**	-5,14	±0,64	-1,85	±0,79	-3,79	±0,02	-6,03	±0,31	-5,46	±0,21	-4,99	±1,29
**Prazosin HCl**	-6,15	±1,71	-2,78	±1,76	-5,05	±1,43	-4,37	±2,50	-4,86	±3,25	-1,57	±0,79
**Pyrithione zinc**	-4,45	±0,35	-3,36	±0,70	-3,34	±0,05	-3,53	±0,21	-5,40	±0,03	-2,71	±1,27
**Quetiapine**	-2,78	±0,62	-2,46	±1,29	-4,34	±1,97	-4,31	±1,05	-3,35	±0,90	-2,13	±0,74
**Rapamycin**	-2,87	±0,40	-1,28	±0,11	-2,64	±0,56	-2,71	±0,08	-3,65	±0,42	-3,41	±0,56
**Sertraline HCl**	-6,04	±0,13	-4,70	±0,22	-4,38	±0,23	-4,62	±0,02	-3,11	±3,87	-1,05	±1,08
**Simvastatin**	-5,20	±0,22	-2,86	±0,10	-2,10	±0,41	-2,08	±0,55	-3,29	±1,03	-3,10	±0,64
**Solifenacin**	-2,14	±0,15	-4,44	±0,68	-3,07	±0,06	-3,03	±0,25	-2,39	±0,42	-3,16	±0,78
**Sorafenib**	-6,14	±0,51	-4,38	±0,38	-5,43	±0,50	-5,76	±0,38	-9,00	±0,72	-5,37	±0,65
**Sulconazole Nitrate**	-2,62	±0,64	-2,59	±1,04	-2,96	±0,18	-6,17	±0,19	-3,27	±1,97	-1,40	±0,01
**Suprofen**	-3,52	±0,50	-1,48	±0,88	-2,25	±0,36	-2,48	±0,28	-3,05	±0,32	-2,52	±0,49
**Tadalafil**	-4,18	±0,68	-1,29	±0,86	-3,10	±0,08	-5,01	±0,23	-4,06	±0,23	-3,29	±0,82
**Teniposide**	-3,79	±0,69	-2,96	±0,29	-3,83	±0,36	-3,55	±0,13	-2,71	±1,74	-2,15	±0,89
**Terfenadine**	-3,31	±3,32	-4,46	±0,57	-4,88	±0,00	-1,58	±0,17	-3,32	±2,92	-2,46	±0,84
**Thioguanine**	-3,22	±0,56	-6,28	±0,72	-6,64	±0,05	-6,93	±0,10	-3,74	±1,38	-2,67	±1,12
**Thioridazine HCl**	-4,37	±0,10	-6,22	±0,46	-6,31	±0,07	-6,29	±0,11	-5,62	±1,40	-2,54	±0,10
**Thiostrepton**	-6,03	±0,46	-3,95	±0,55	-4,79	±0,24	-4,93	±0,24	-5,11	±2,64	-0,86	±1,43
**Thonzonium HBr**	-5,07	±0,16	-3,43	±0,49	-3,90	±0,01	-3,98	±0,15	-2,62	±3,62	-0,15	±1,48
**Tioconazole**	-3,39	±0,08	-2,84	±0,18	-4,11	±0,08	-6,41	±0,32	-4,35	±0,03	-3,63	±1,65
**Topotecan HCl**	-6,03	±0,35	-3,78	±0,50	-4,08	±0,16	-4,63	±0,10	-5,79	±0,69	-2,17	±0,70
**Toremifene Citrate**	-3,41	±0,30	-5,37	±0,60	-3,71	±0,23	-4,18	±0,51	-3,42	±0,20	-3,10	±0,71
**Trifluridine**	-4,95	±0,06	-3,31	±0,36	-3,65	±0,42	-4,13	±0,11	-4,86	±0,91	-3,07	±1,13
**Vincristine**	-3,29	±0,06	-2,07	±0,04	-2,32	±0,12	-3,27	±0,04	-2,58	±0,02	-0,96	±0,48
**Vinorelbine**	-3,90	±0,04	-2,87	±0,09	-3,51	±0,02	-4,17	±0,25	-4,09	±0,09	-2,74	±1,56
**Vorinostat**	-7,30	±0,90	-5,66	±0,08	-5,23	±0,38	-5,74	±0,37	-9,46	±0,36	-5,02	±1,09

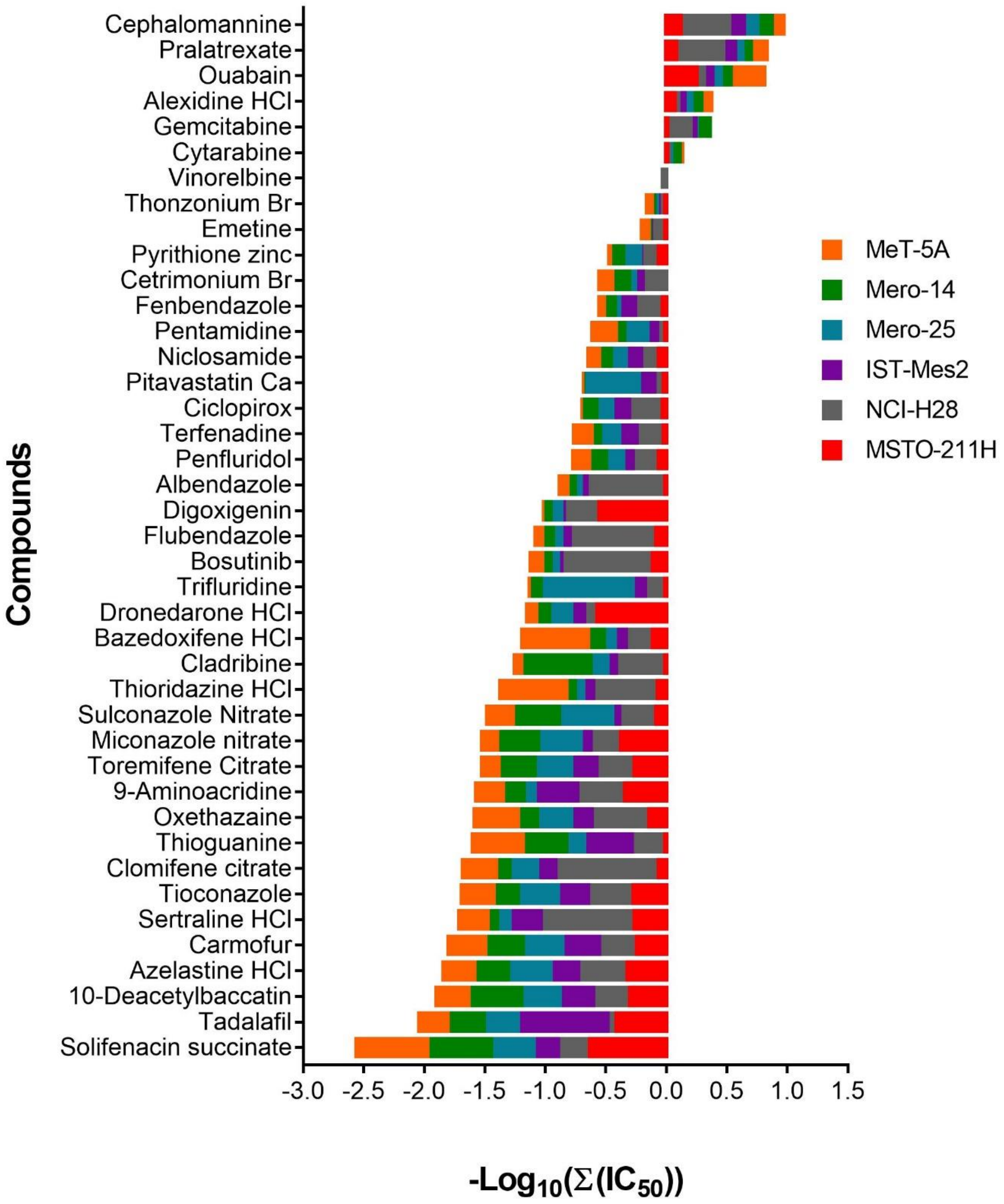

Figure S1. IC_{50}-based ranking of drugs assessed by dose-response cytotoxicity assays. The graph shows the calculation of the -$Log_{10}(\sum(IC_{50}))$ as measure of the overall cytotoxic activity of 41 compounds out of 42 undergone to validation on an extended dose range. The colours represent the extent of response of each cell line. Allylthiourea is not reported because it did not confirm its toxicity and the calculation of the IC_{50} was not possible. Drugs were ranked on the Y-axis, from the top to the bottom, based on a decreasing activity. IC_{50} values were calculated for each compound, in each cell line, employing the validation curves reported in Appendix A.

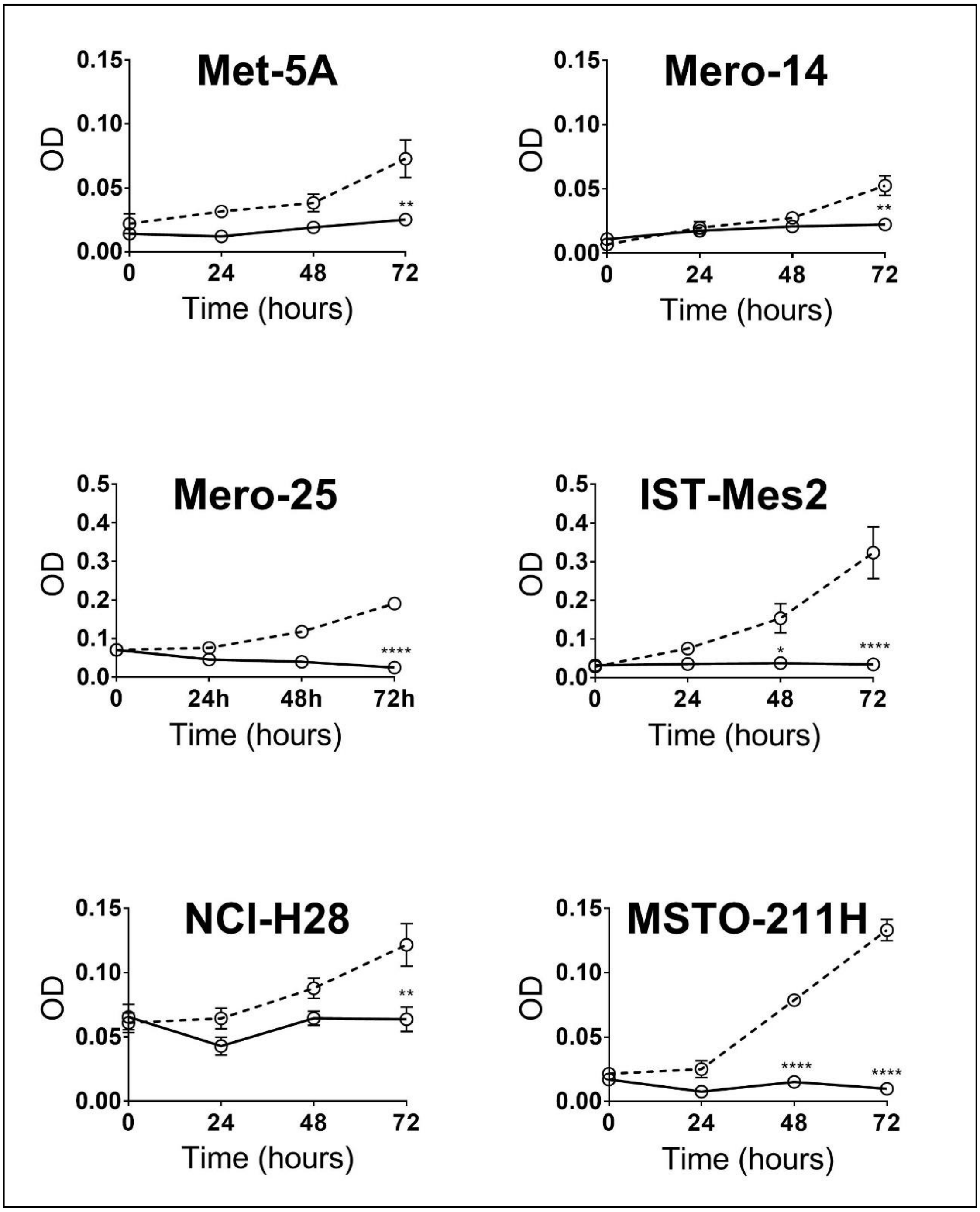

Figure S2. **MTT Assay for CM.** Proliferation of mesothelial (MeT-5A) and MPM cells lines (Mero-14, Mero-25, IST-Mes2, MSTO-211H, and NCI H28) after treatment with CM 0.1 µM (solid line), expressed as number of vital cells evaluated with the MTT assay. Dotted line refers to the control group treated with DMSO. Y-axis reports mean ± SEM of the OD measured at 595nm from three independent experiments, each performed in triplicate. X-axis reports the hours from the moment of treatment. Statistical significance is indicated by asterisks (*), where * = P <0.05; ** = P <0.01; *** = P <0.001, and **** = P <0.0001 with respect to each control curve.

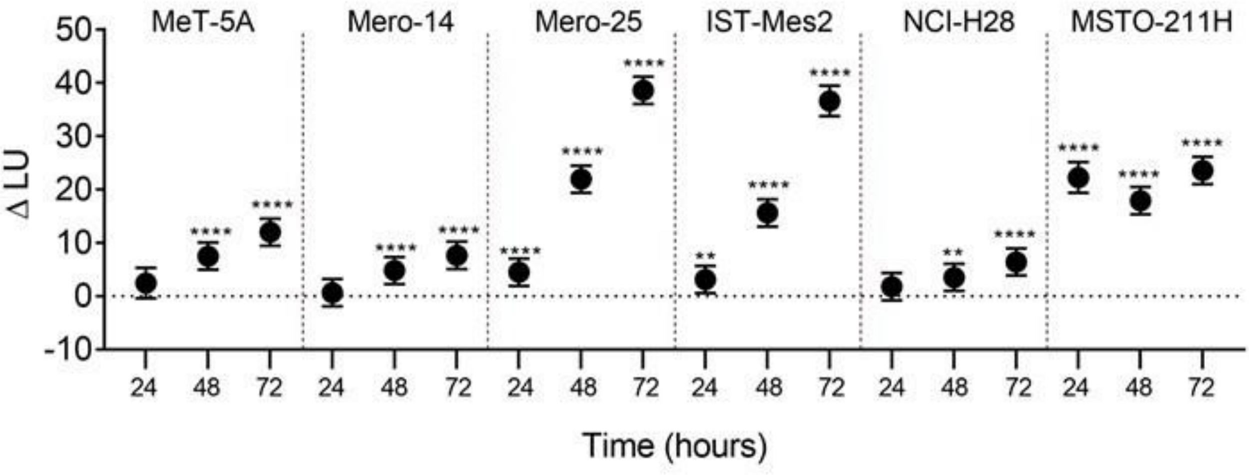

A

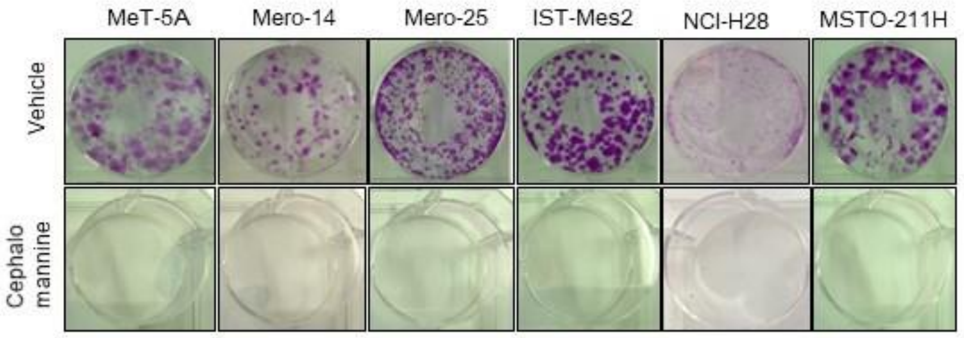

B

Figure S3. Caspases and CFA assays after treatment with CM at 0.1µM in MeT-5A mesothelial cell line and in Mero-14, Mero-25, IST-Mes2, NCI-H28, and MSTO-211H MPM cell lines. (A) Y-axis reports the average difference (ΔLU) (as dots) and its confidence interval (as whiskers) between the units of luminescence obtained with the Caspase-Glo® 3/7 assay and measured after 24, 48, and 72 hours in treated and control cultures in three independent experiments. X-axis reports the employed cell lines and the time. When the differences were statistically significant, their divergence from 0 were represented by asterisks, as * = P <0.05; ** = P <0.01; *** = P <0.001 and **** = P <0.0001 **(B)** The representative picture, for visual purposes only, shows the colonies obtained after 11 days of the treatment with CM, in the different cell lines. Controls consist in a treatment with the vehicle only (DMSO).

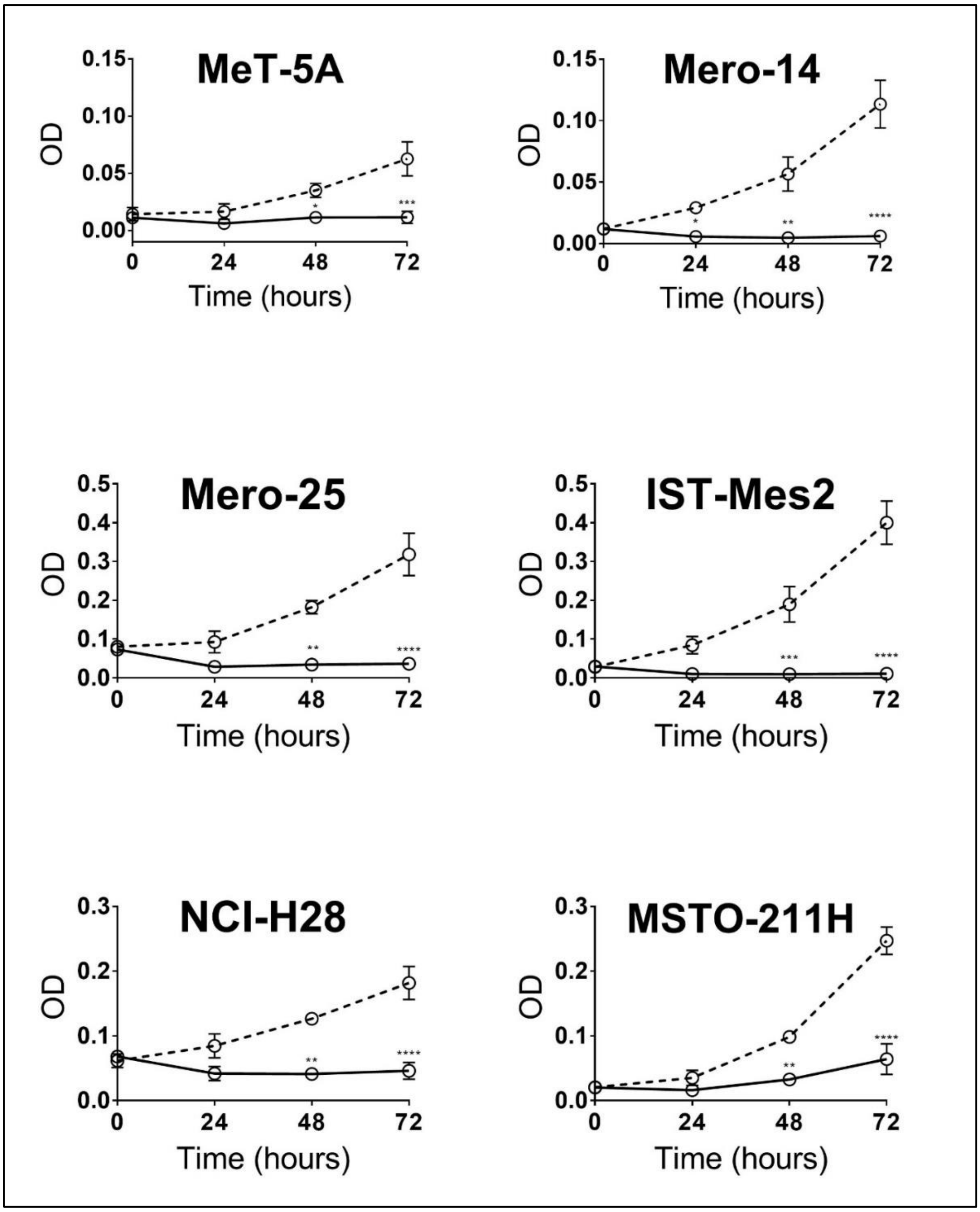

Figure S4. MTT Assay for OB. Proliferation of mesothelial (MeT-5A) and MPM cells lines (Mero-14, Mero-25, IST-Mes2, MSTO-211H, and NCI H28) after treatment with OB 0.1 µM (solid line), expressed as number of vital cells evaluated with the MTT assay. Dotted line refers to the control group treated with DMSO. Y-axis reports mean ± SEM of the OD measured at 595nm from three independent experiments, each performed in triplicate. X-axis reports the hours from the moment of treatment. Statistical significance is indicated by asterisks (*), where * = P <0.05; ** = P <0.01; *** = P <0.001, and **** = P <0.0001 with respect to each control curve.

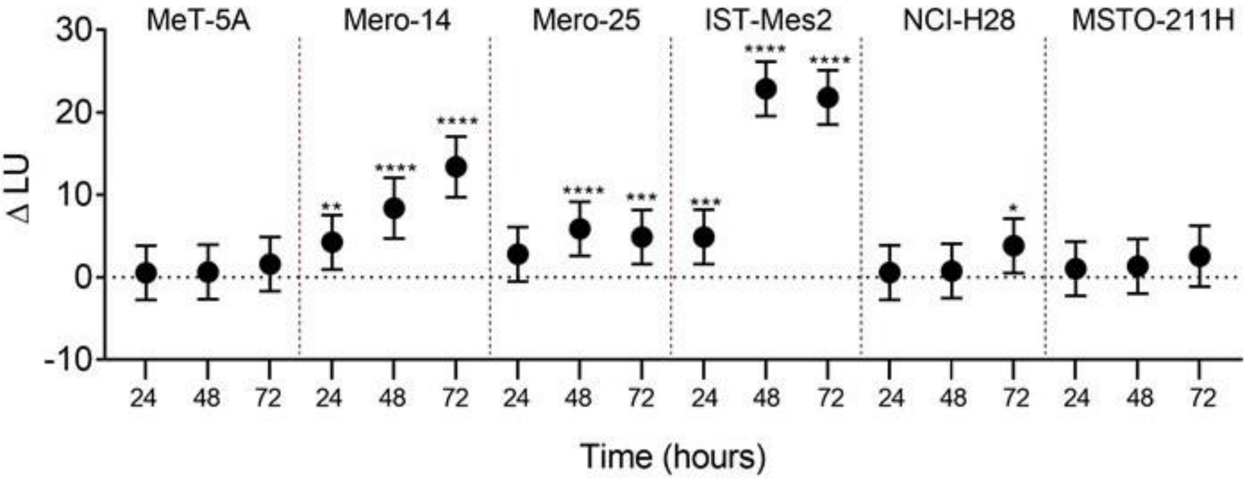

A

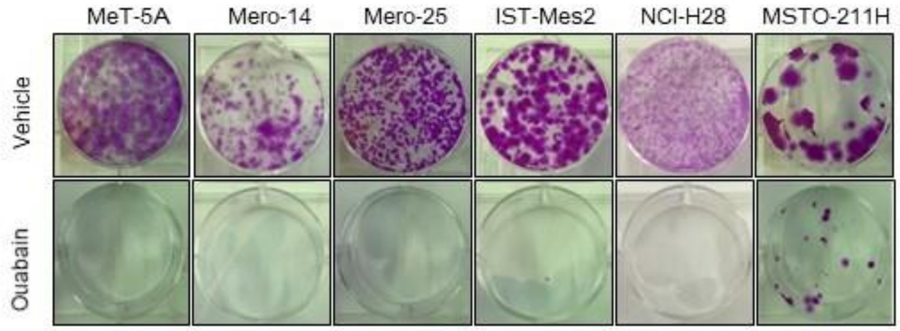

B

Figure S5. Caspases and CFA assays after treatment with OB at 0.1μM in MeT-5A mesothelial cell line and in Mero-14, Mero-25, IST-Mes2, NCI-H28, and MSTO-211H MPM cell lines. (A) Y-axis reports the average difference (ΔLU) (as dots) and its confidence interval (as whiskers) between the units of luminescence obtained with the Caspase-Glo® 3/7 assay and measured after 24, 48, and 72 hours in treated and control cultures in three independent experiments. X-axis reports the employed cell lines and the time. When the differences were statistically significant, their divergence from 0 were represented by asterisks, as * = $P < 0.05$; ** = $P < 0.01$; *** = $P < 0.001$ and **** = $P < 0.0001$ **(B)** The representative picture, for visual purposes only, shows the colonies obtained after 11 days of the treatment with OB, in the different cell lines. Controls consist in a treatment with the vehicle only (DMSO).

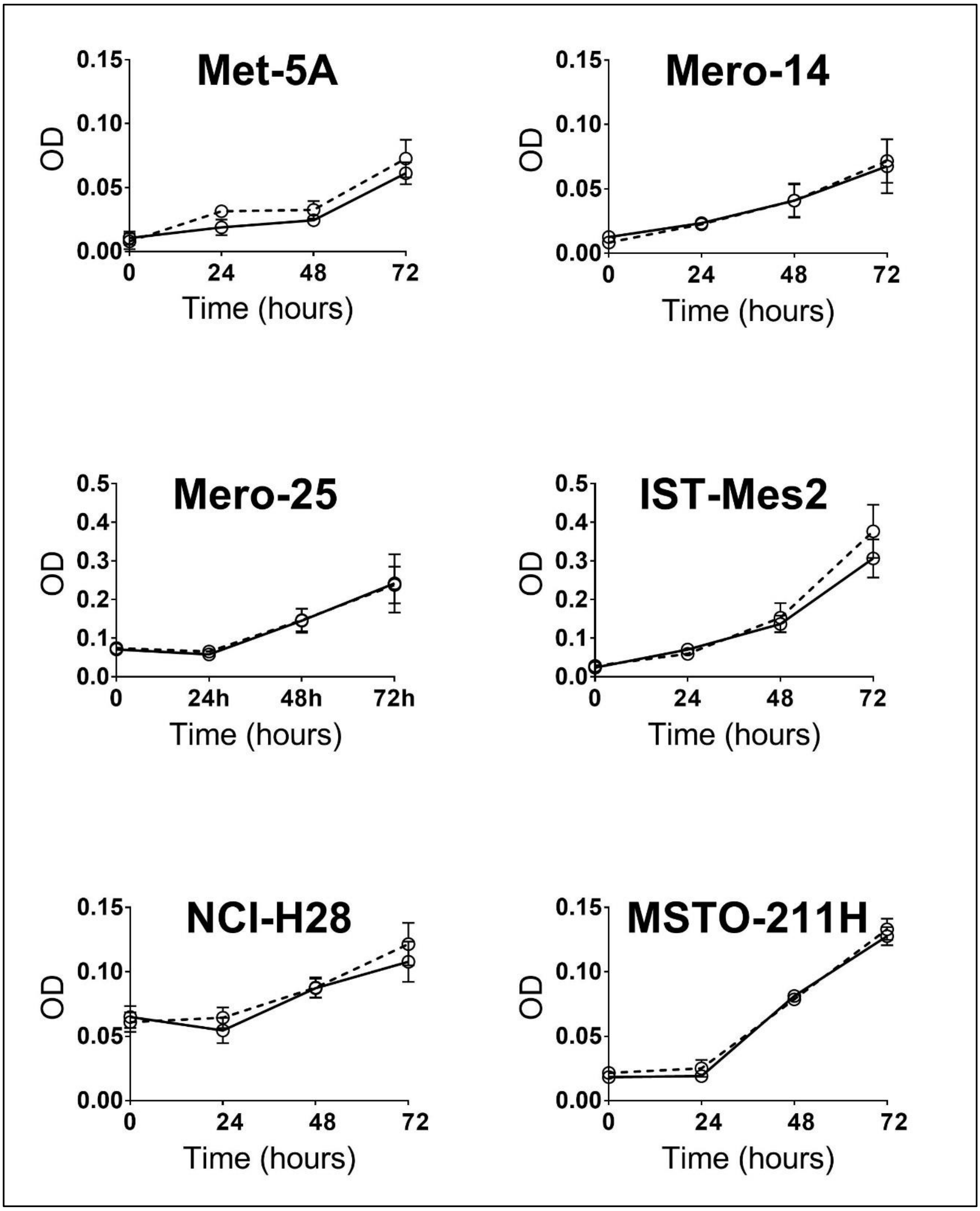

Figure S6. MTT Assay and AX. Proliferation of the mesothelial cell line MeT-5A and of the MPM lines (Mero-14, Mero-25, IST-Mes2, MSTO-211H, and NCI H28) after treatment with AX 0.1 μM (solid line), expressed as number of vital cells evaluated with the MTT assay. Dotted line refers to the control group treated with DMSO. Y-axis reports mean ± SEM of the OD measured at 595nm from three independent experiments, each performed in triplicate. X-axis reports the hours from the moment of treatment. Statistical significance is indicated by asterisks (*), where * = $P < 0.05$; ** = $P < 0.01$; *** = $P < 0.001$, and **** = $P < 0.0001$ with respect to each control curve.

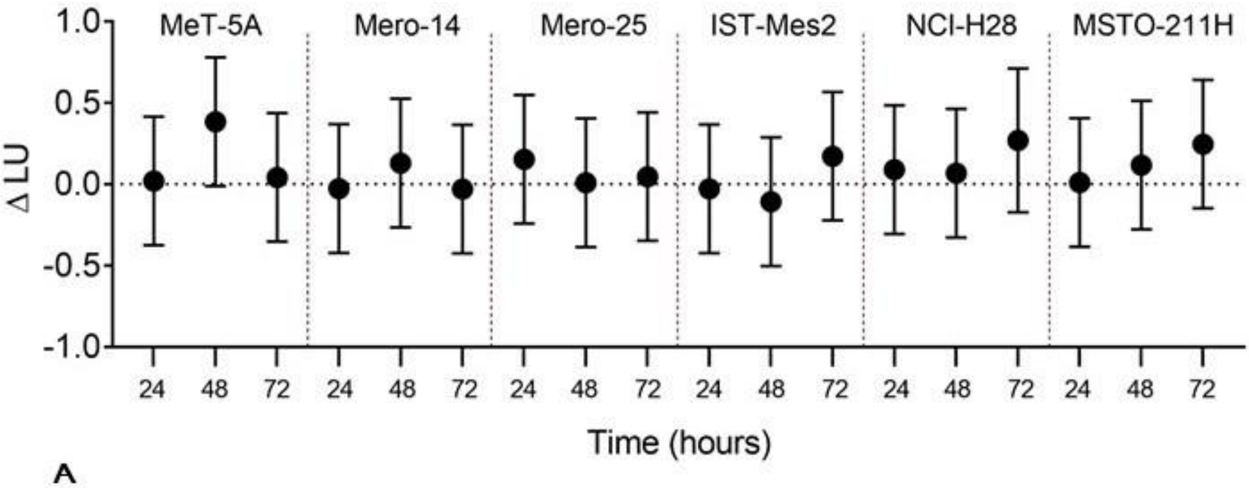

A

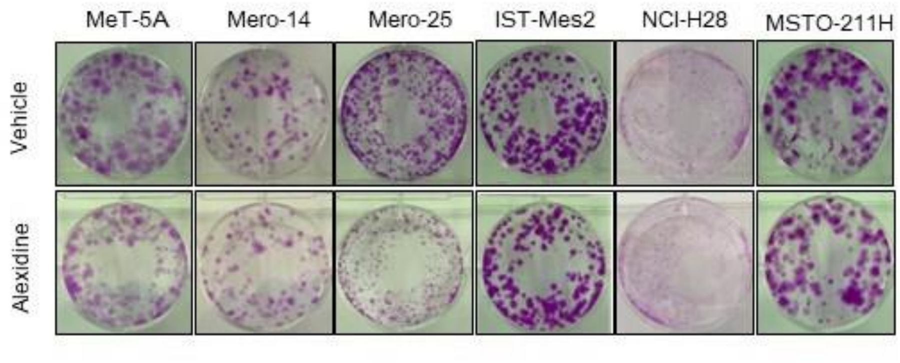

B

Figure S7. Caspases and CFA assays after treatment with AX at 0.1µM in MeT-5A mesothelial cell line and in Mero-14, Mero-25, IST-Mes2, NCI-H28, and MSTO-211H MPM cell lines.

(A) Y-axis reports the average difference (ΔLU) (as dots) and its confidence interval (as whiskers) between the units of luminescence obtained with the Caspase-Glo® 3/7 assay and measured after 24, 48, and 72 hours in treated and control cultures in three independent experiments. X-axis reports the employed cell lines and the time. When the differences were statistically significant, their divergence from 0 were represented by asterisks, as * = P <0.05; ** = P <0.01; *** = P <0.001 and **** = P <0.0001 **(B)** The representative picture, for visual purposes only, shows the colonies obtained after 11 days of the treatment with AX, in the different cell lines. Controls consist in a treatment with the vehicle only (DMSO).

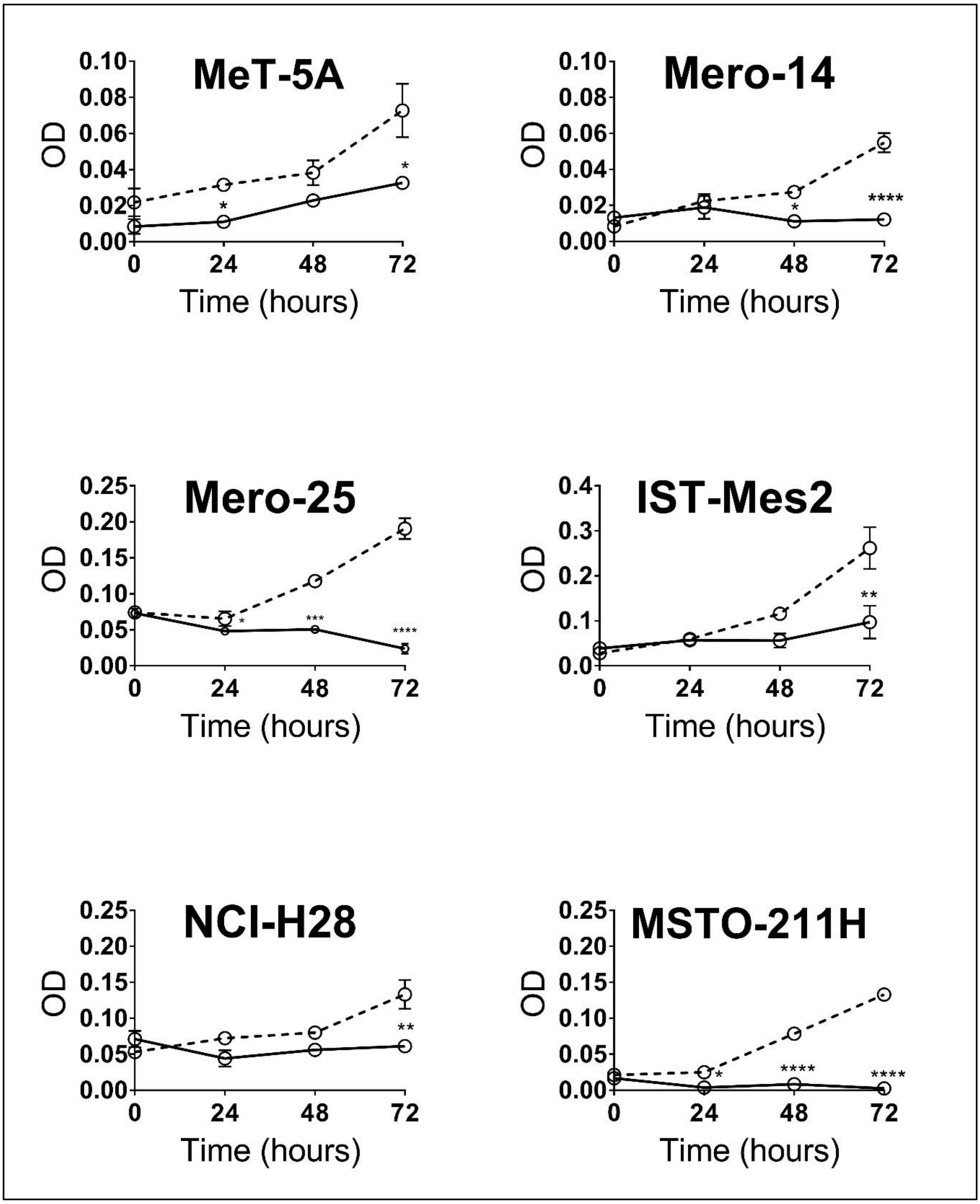

Figure S8. **MTT Assay for TB**. Proliferation of the mesothelial cell line MeT-5A and of the MPM lines (Mero-14, Mero-25, IST-Mes2, MSTO-211H, and NCI H28) after treatment with TB 1 µM (solid line), expressed as number of vital cells evaluated with the MTT assay. Dotted line refers to the control group treated with DMSO. Y-axis reports mean ± SEM of the OD measured at 595nm from three independent experiments, each performed in triplicate. X-axis reports the hours from the moment of treatment. Statistical significance is indicated by asterisks (*), where * = P <0.05; ** = P <0.01; *** = P <0.001, and **** = P <0.0001 with respect to each control curve.

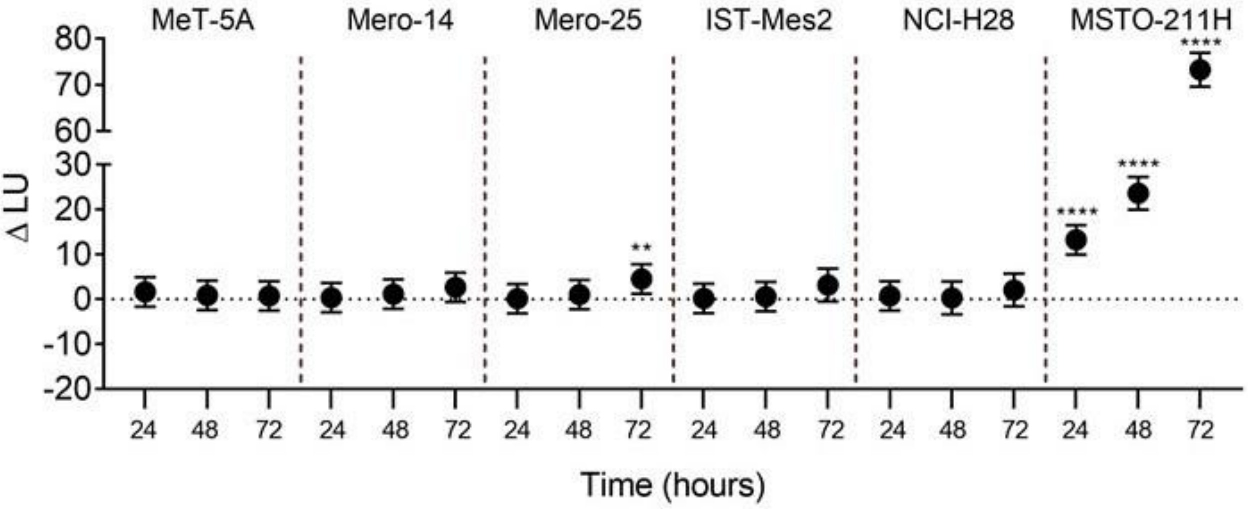

A

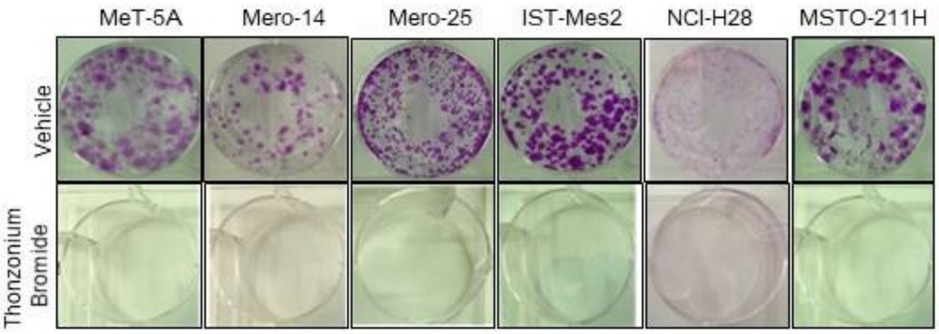

B

Figure S9. Caspases and CFA assays after treatment with TB at 1μM in MeT-5A mesothelial cell line and in Mero-14, Mero-25, IST-Mes2, NCI-H28, and MSTO-211H MPM cell lines. (A) Y-axis reports the average difference (ΔLU) (as dots) and its confidence interval (as whiskers) between the units of luminescence obtained with the Caspase-Glo® 3/7 assay and measured after 24, 48, and 72 hours in treated and control cultures in three independent experiments. X-axis reports the employed cell lines and the time. When the differences were statistically significant, their divergence from 0 were represented by asterisks, as * = P <0.05; ** = P <0.01; *** = P <0.001 and **** = P <0.0001 **(B)** The representative picture, for visual purposes only, shows the colonies obtained after 11 days of the treatment with TB, in the different cell lines. Controls consist in a treatment with the vehicle only (DMSO).

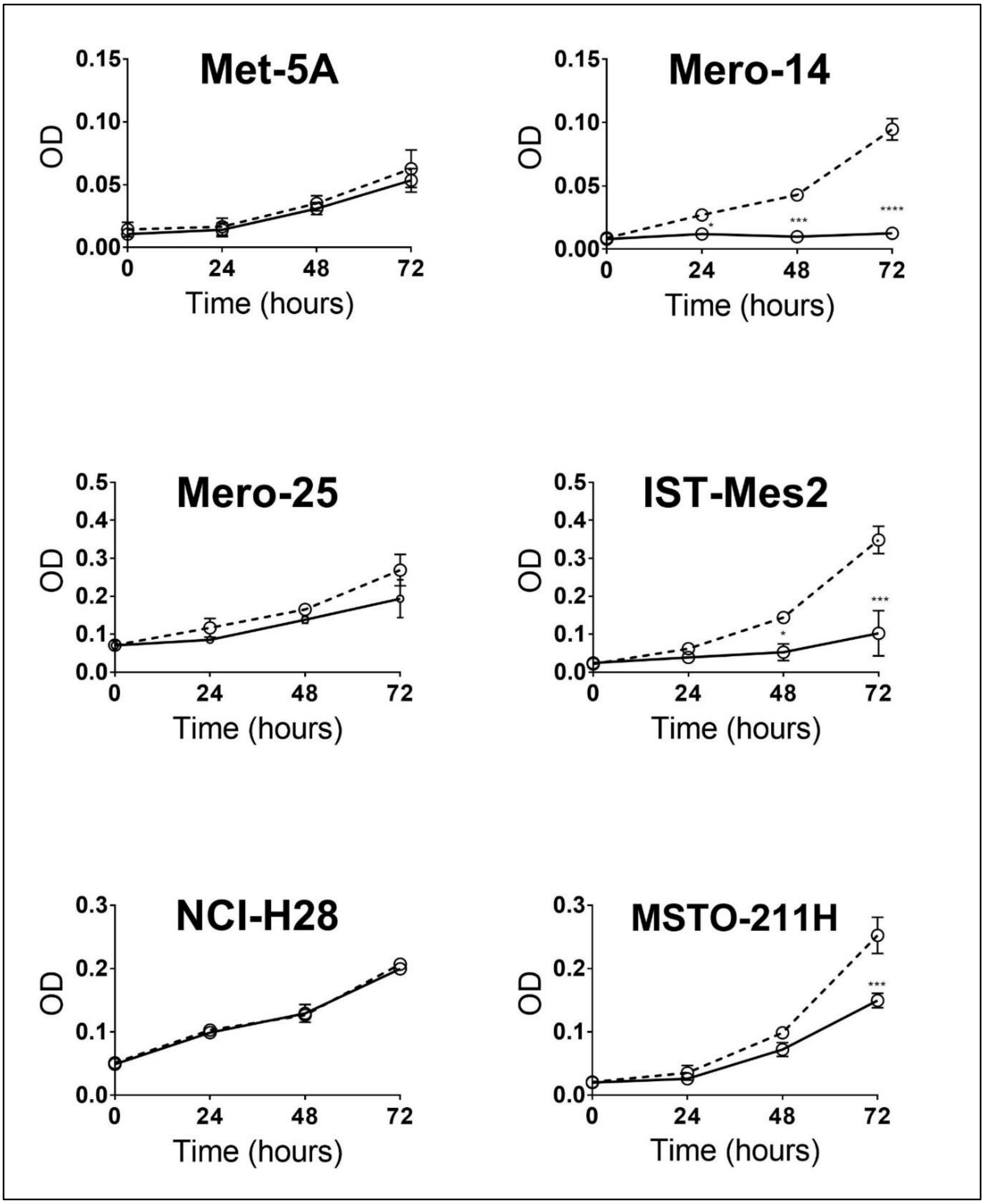

Figure S10. MTT Assay for EM. Proliferation of the mesothelial cell line MeT-5A and of the MPM lines (Mero-14, Mero-25, IST-Mes2, MSTO-211H, and NCI H28) after treatment with EM 0.1 µM, expressed as number of vital cells evaluated with the MTT assay. Y-axis reports mean ± SEM of the OD measured at 595nm from three independent experiments, each performed in triplicate. X-axis reports the hours from the moment of treatment.Statistical significance is indicated by asterisks (*), where * = $P < 0.05$; ** = $P < 0.01$; *** = $P < 0.001$, and **** = $P < 0.0001$ with respect to each control curve.

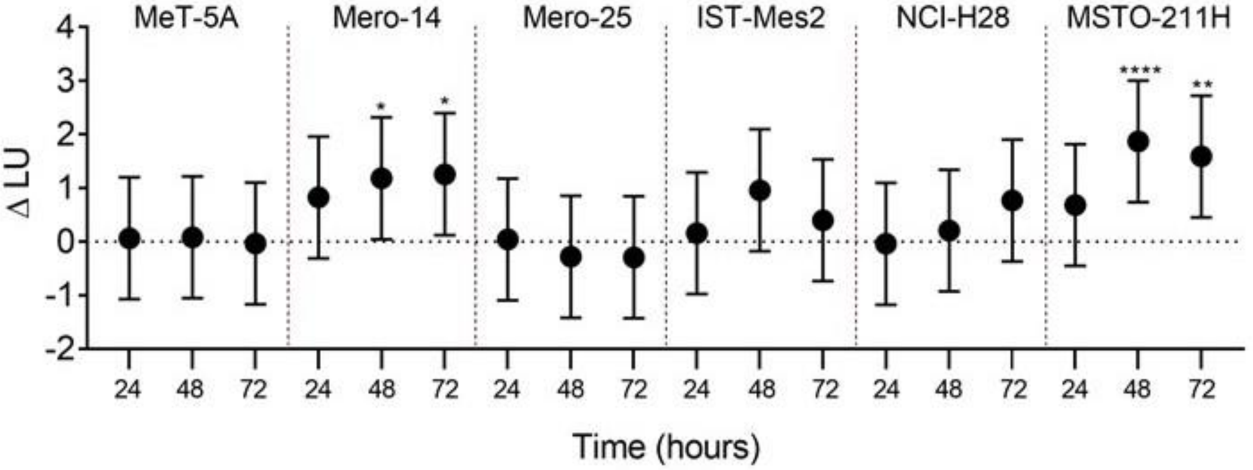

A

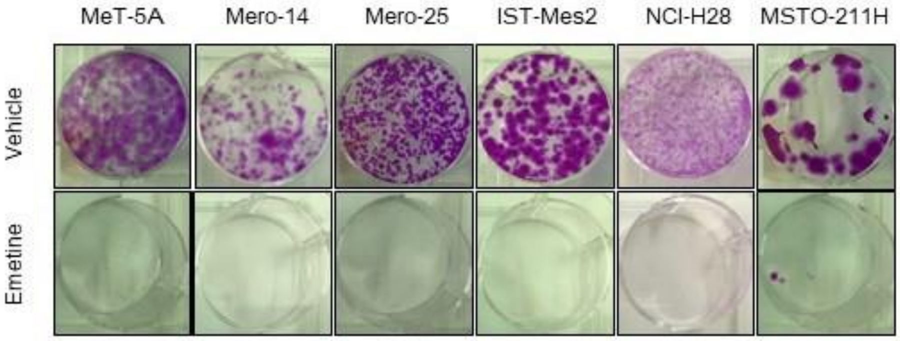

B

Figure S11. Caspases and CFA assays after treatment with EM at 0.1µM in MeT-5A mesothelial cell line and in Mero-14, Mero-25, IST-Mes2, NCI-H28, and MSTO-211H MPM cell lines.

(A) Y-axis reports the average difference (ΔLU) (as dots) and its confidence interval (as whiskers) between the units of luminescence obtained with the Caspase-Glo® 3/7 assay and measured after 24, 48, and 72 hours in treated and control cultures in three independent experiments. X-axis reports the employed cell lines and the time. When the differences were statistically significant, their divergence from 0 were represented by asterisks, as * = P <0.05; ** = P <0.01; *** = P <0.001 and **** = P <0.0001. **(B)** The representative picture, for visual purposes only, shows the colonies obtained after 11 days of the treatment with EM, in the different cell lines. Controls consist in a treatment with the vehicle only (DMSO).

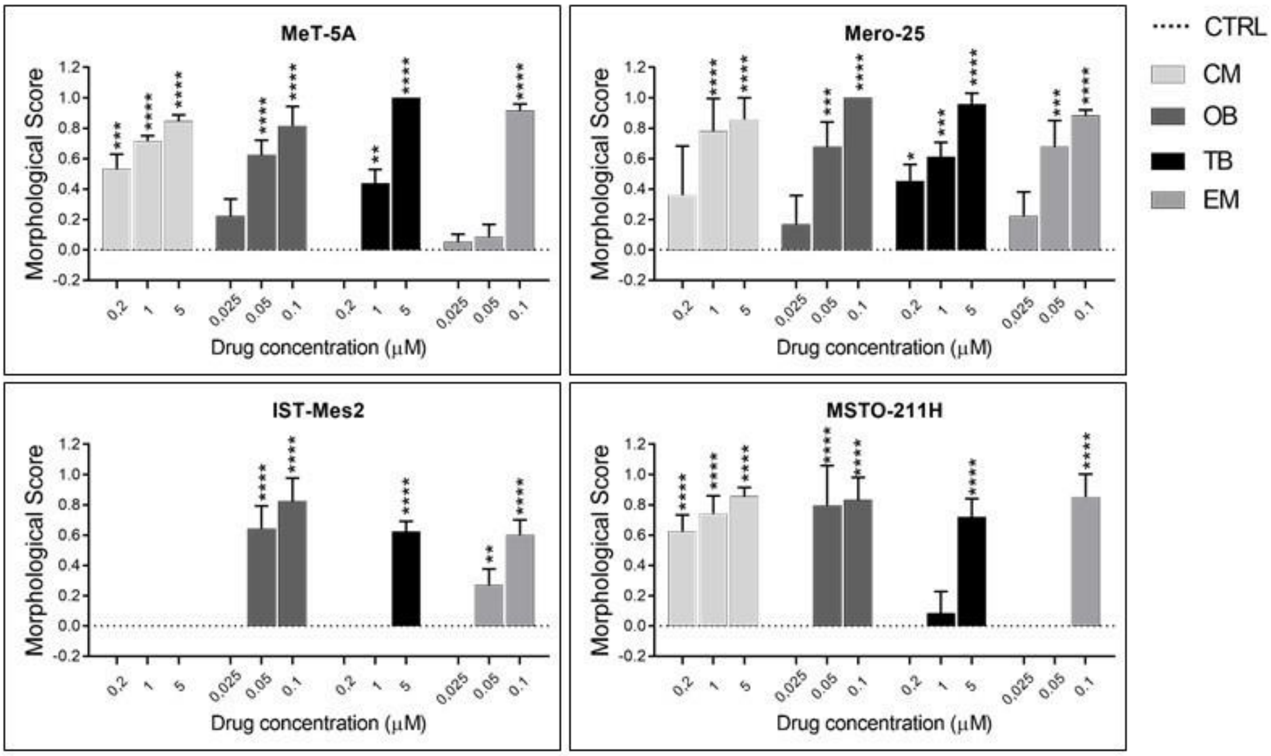

Figure S12. Spheroid assay on MeT-5A mesothelial cell line and Mero-25, IST-Mes2 and MSTO-211H MPM cell lines. Excess share of spheroids affected by morphological changes after CM, OB, TB, or EM treatment. For each cell line, values obtained in the untreated cultures were subtracted and used as reference (dotted line). The scores are represented as mean ± SEM of three experiments obtained by two independent readers blinded for the state of the spheroids. The statistical significance is marked by asterisks (*), where * = P <0.05; ** = P <0.01; *** = P <0.001, and ****.= P <0.0001

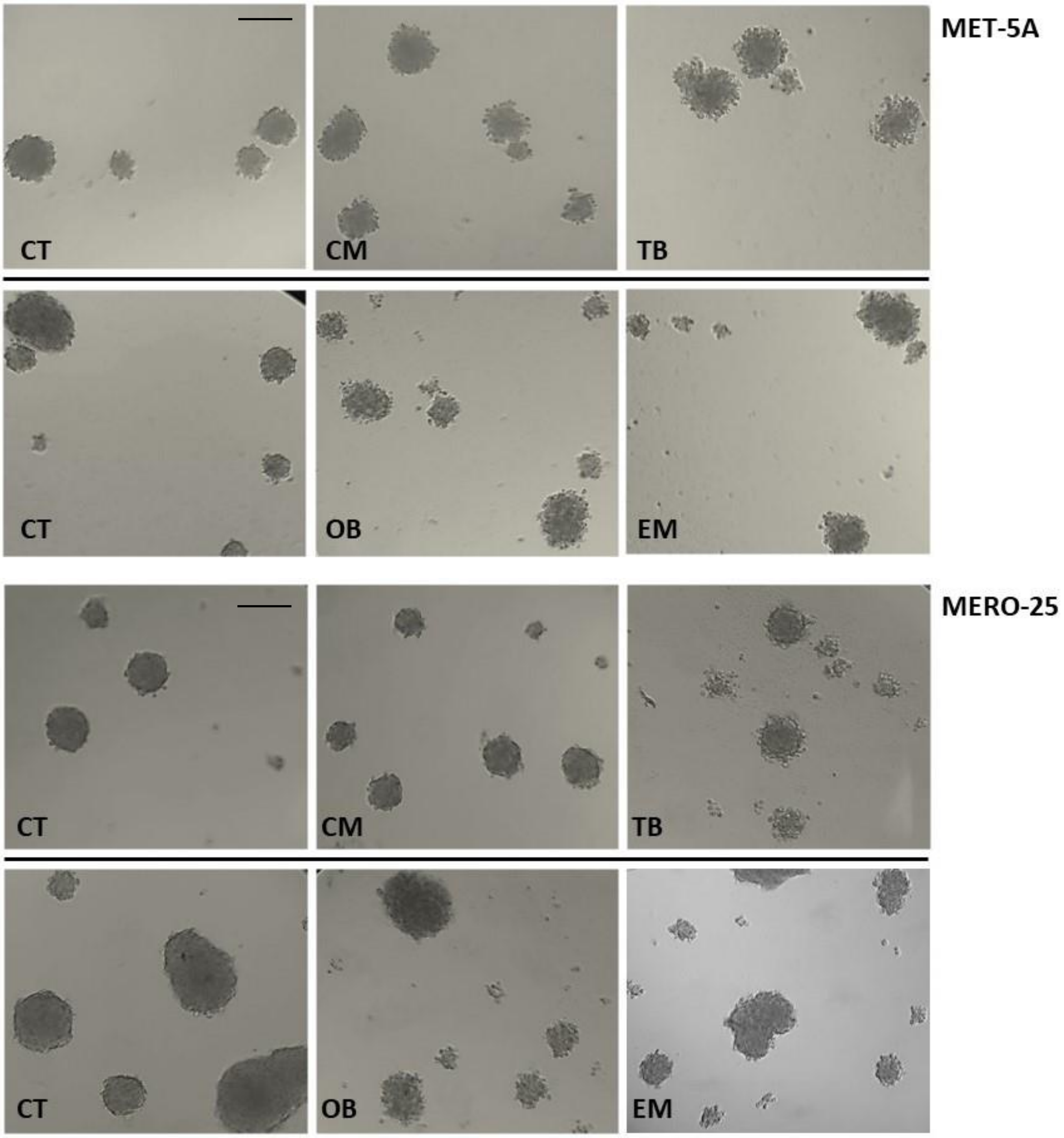
MET-5A
CT
CM
TB
CT
OB
EM
MERO-25
CT
CM
TB
CT
OB
EM

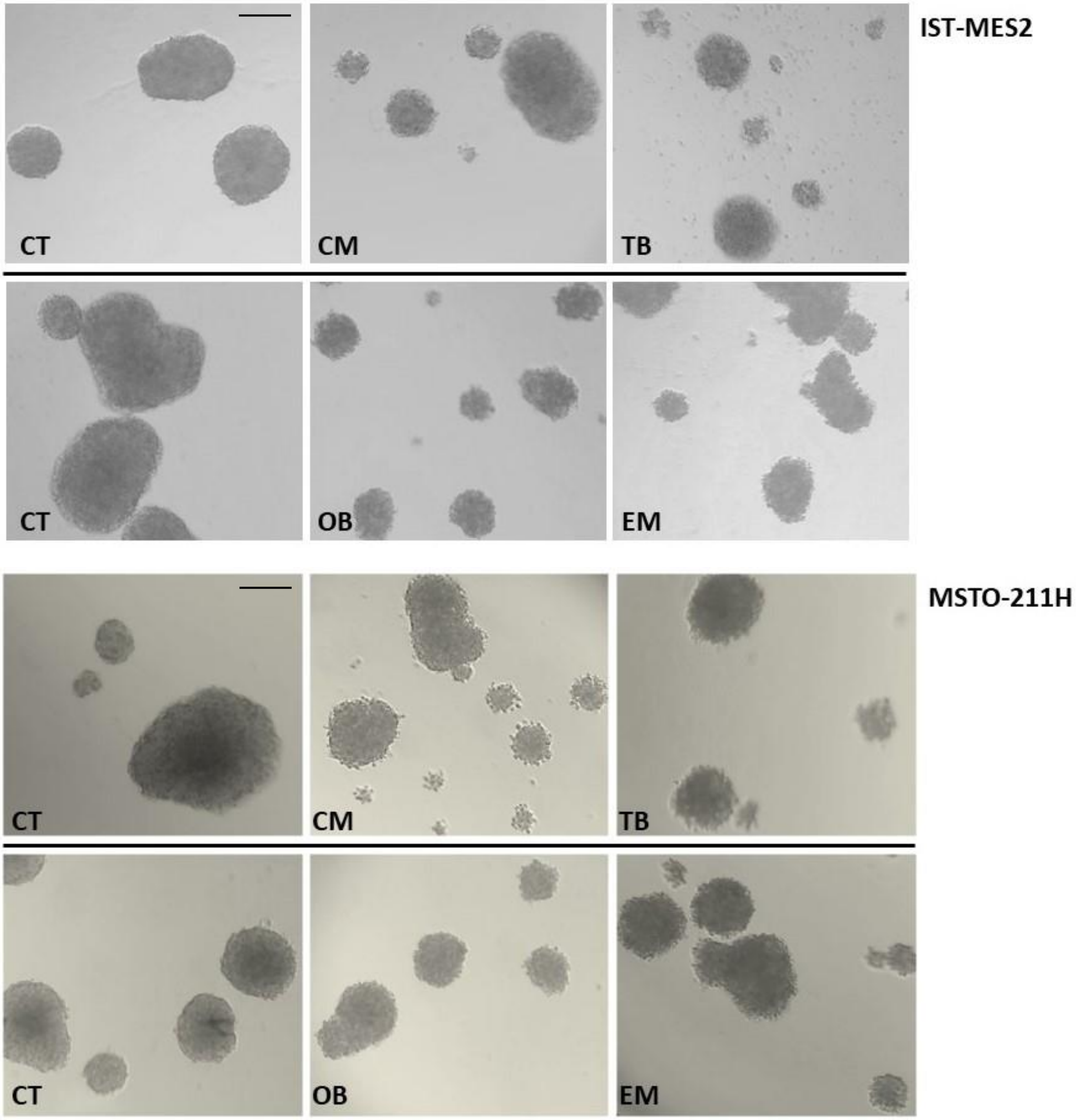

Figure S13. Spheroid assay on MeT-5A and Mero-25, IST-Mes2, and MSTO-211H MPM cells. Representative images of spheroid cultures of MeT-5A and MPM cells, after 72 hours of treatment with CM 5 µM, TB 5 µM, OB 1 µM, EM 1 µM or vehicle (DMSO for CM and TB, H_20 for OB and EM). Spheroid images in suspension culture were captured at 10X magnification. Scale bar = 100 µm

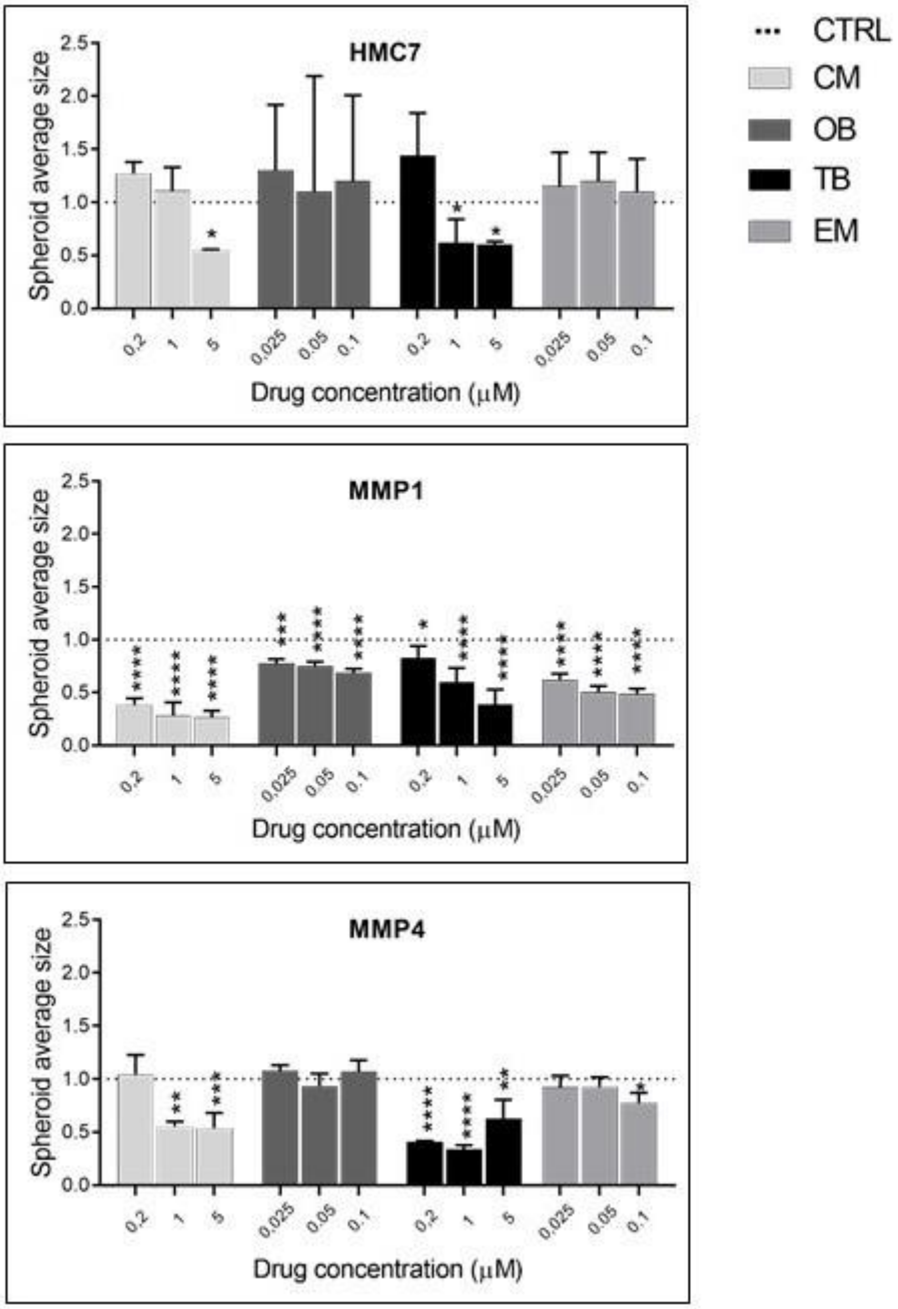

Figure S14. Spheroid assay on patient non-immortalized HMC7 and tumor MMP1 and MMP4 cells. Graphs showing the relative average size formed by HMC7, MMP1 and MMP4 cells after CM, OB, TB or EM treatment, related to the control (dotted line). Y-axis reports the spheroids relative size as the average number of pixels of the occupied area, measured with the software ImageJ by two independent readers blinded for the state of the spheroids. Data are reported as mean ± SEM of two experiments, each performed in duplicate. The statistical significance is indicated by asterisks (*), where * = P <0.05; ** = P <0.01; *** = P <0.001, and **** = P <0.0001.

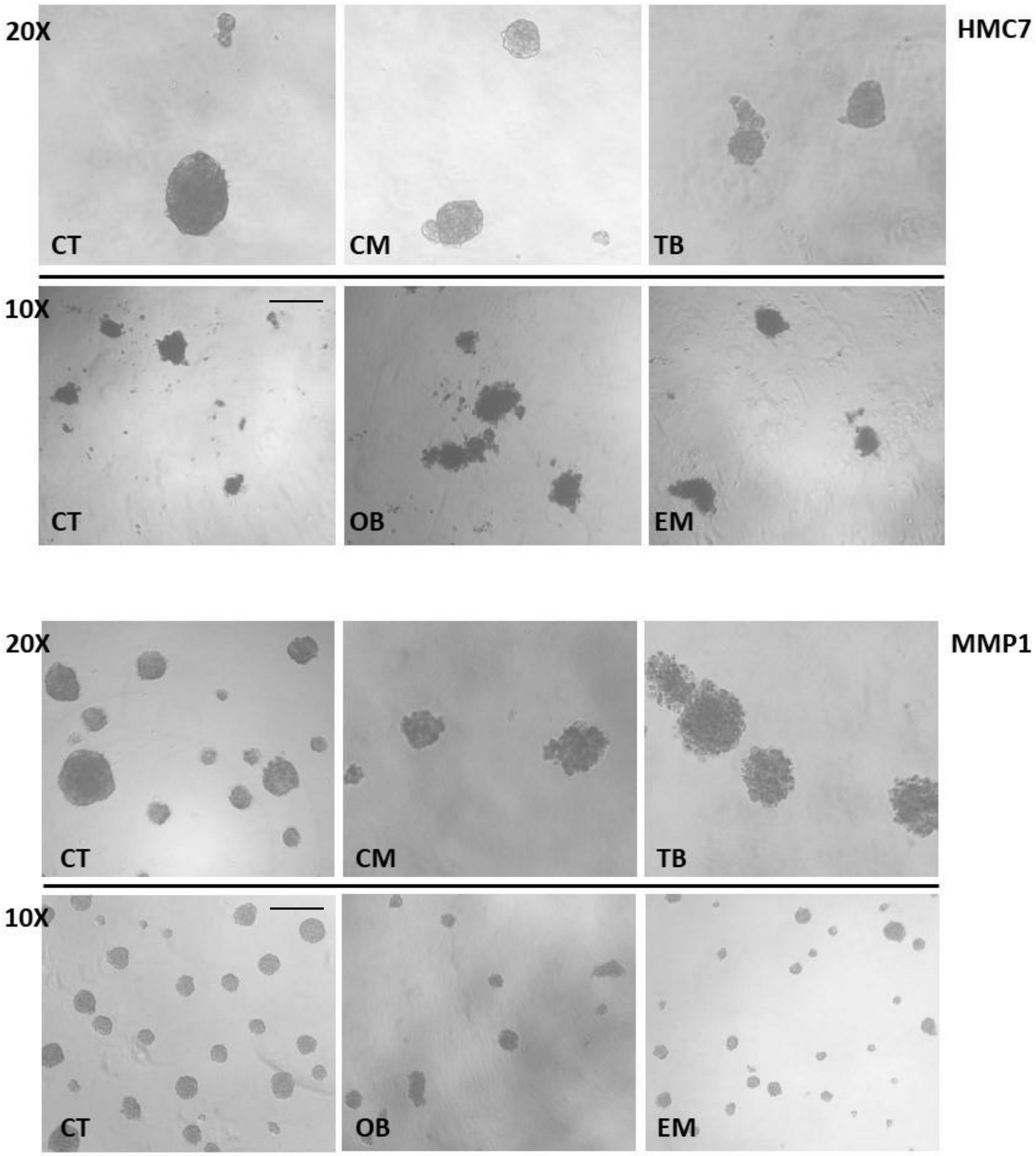
20X
HMC7
CT
CM
TB
10X
CT
OB
EM
20X
MMP1
CT
CM
TB
10X
CT
OB
EM

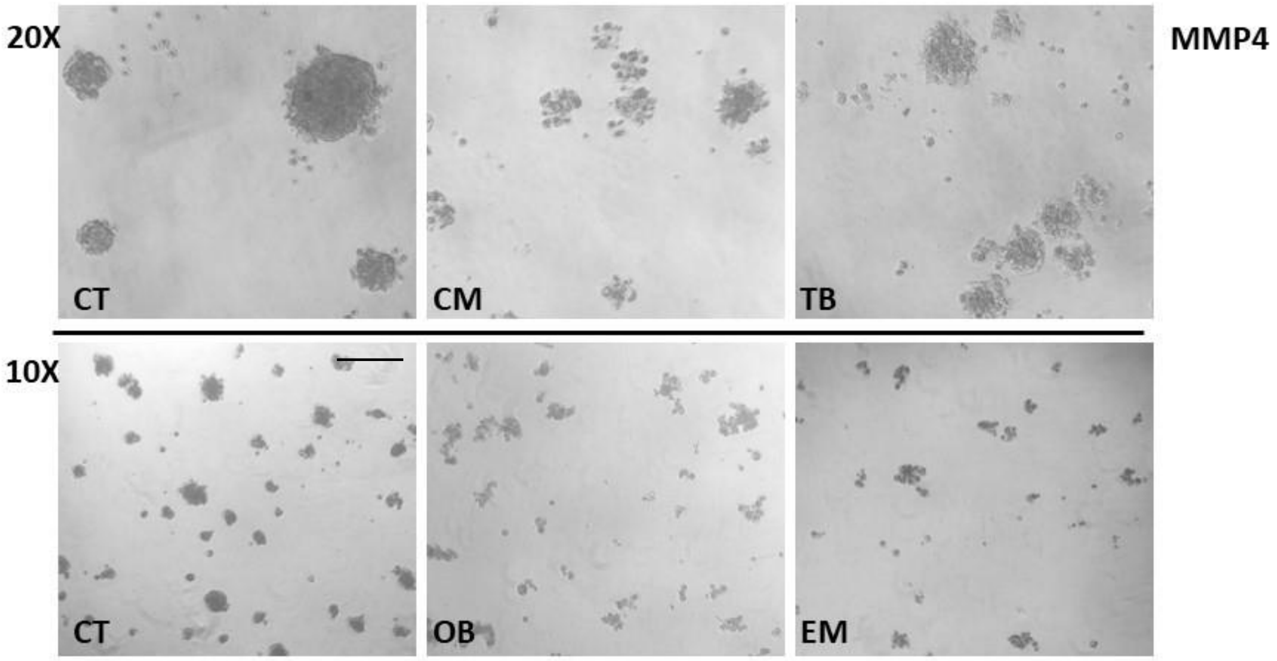

Figure S15. Spheroid assay on patient non-immortalized HMC7 and tumor MMP1 and MMP4 cells. Representative images of spheroid cultures of HMC7, MMP1 and MMP4 cells, after 72 hours of treatment with CM 5 µM, TB 5 µM, OB 1 µM, EM 1 µM or vehicle (DMSO for CM and TB, H_20 for OB and EM). Spheroid images in suspension culture were captured at either 10X or 20X magnification. Scale bar = 100 µm.
