# Supplementary material for: A Drug Screening Revealed Novel Potential Agents against Malignant Pleural Mesothelioma
Source: Cancers (Basel). 2022 May 20;14(10):2527. doi: 10.3390/cancers14102527 (PMC9139775; doi:10.3390/cancers14102527)

10-Deacetylbaccatin

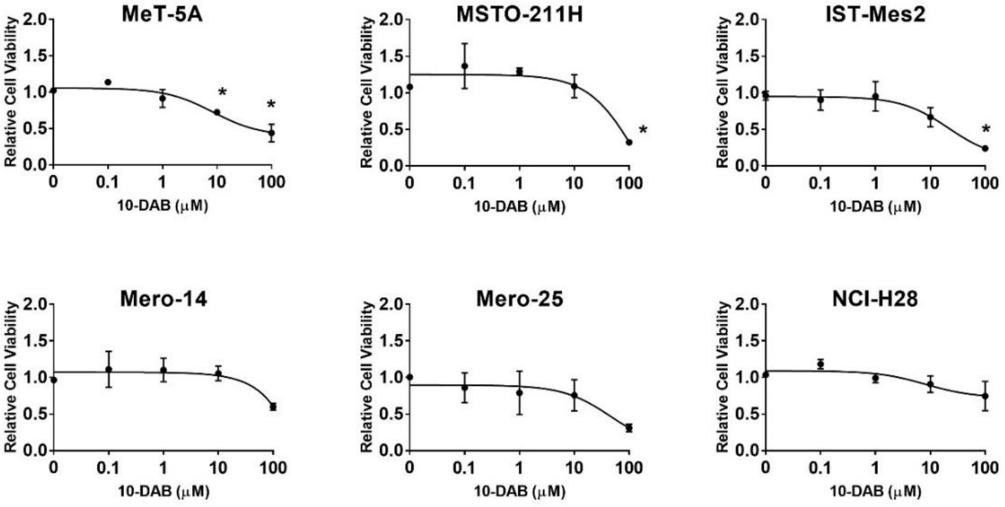

9-Aminoacridine

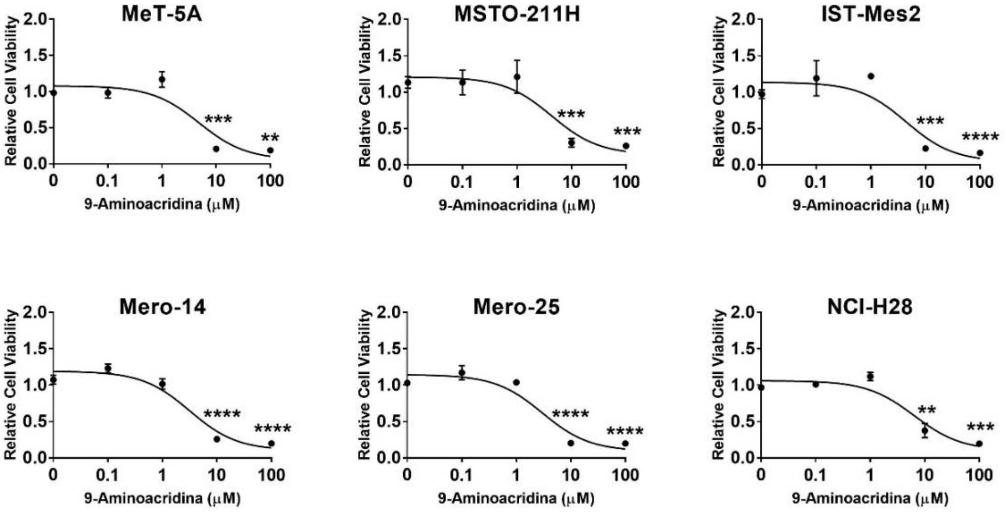

Albendazole

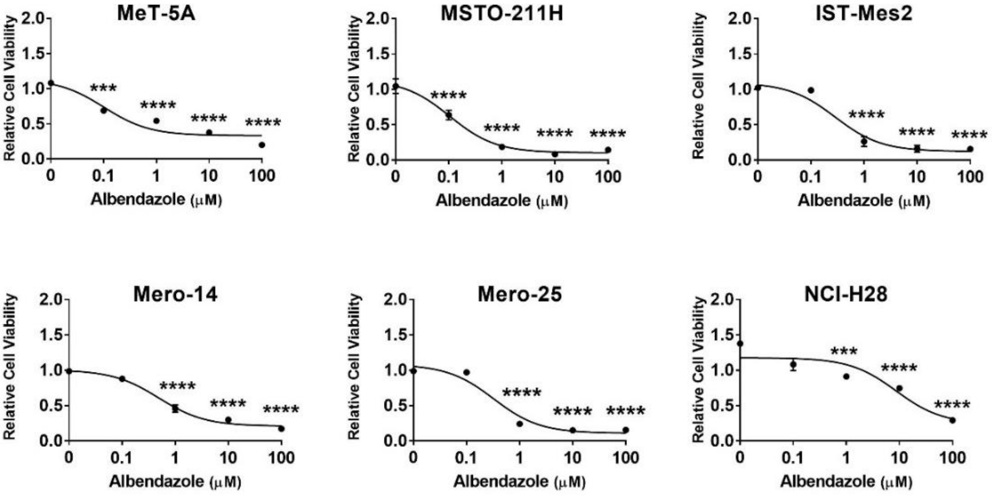

Alexidine

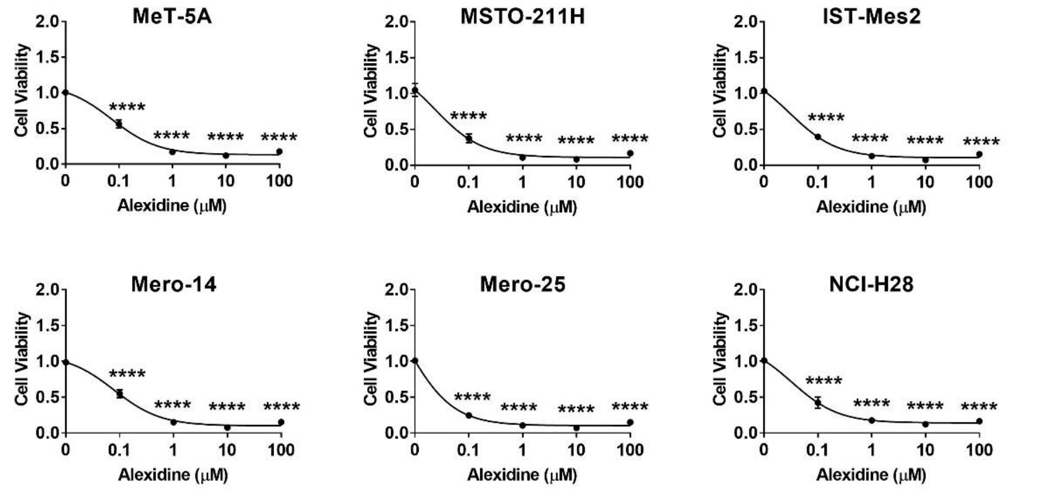

Allylthiourea

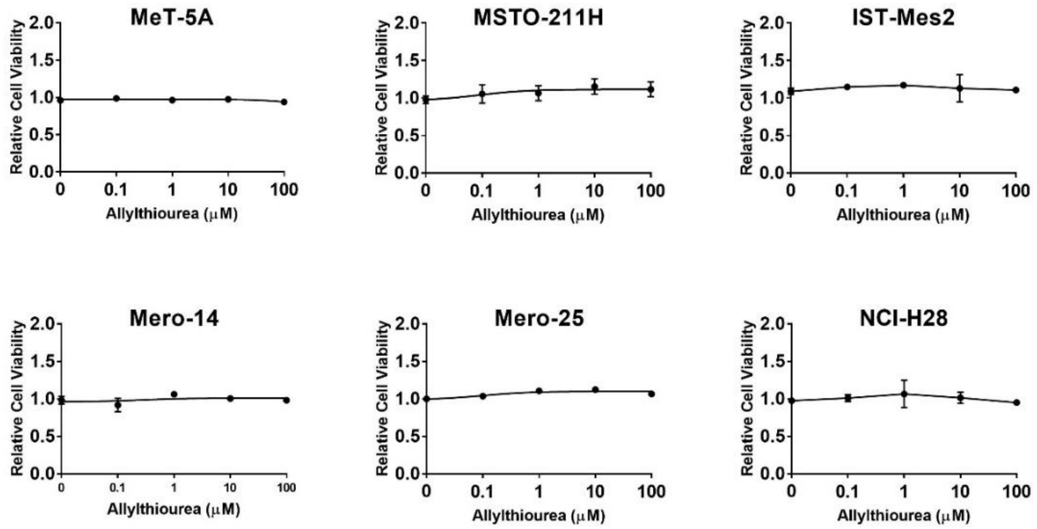

Azelastine hydrochloride

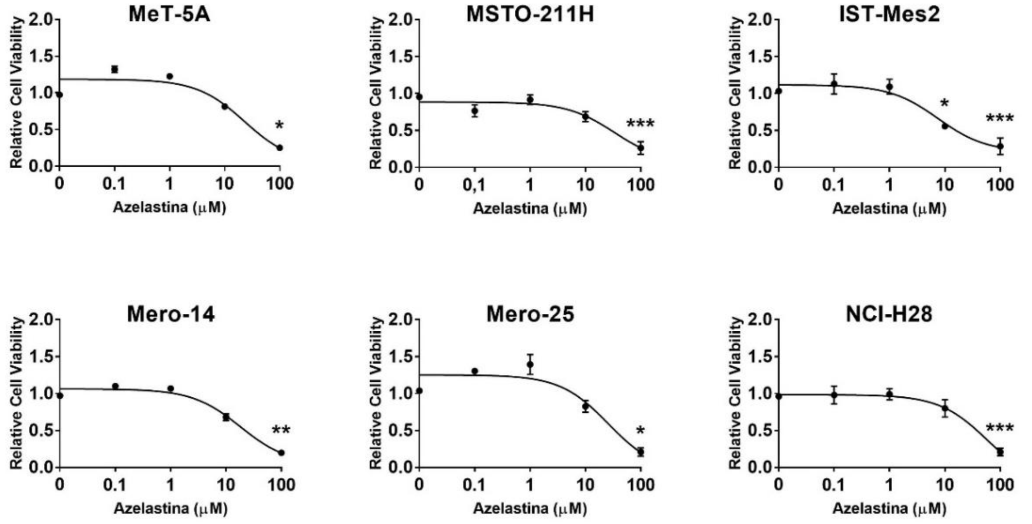

Bazedoxifene hydrochloride

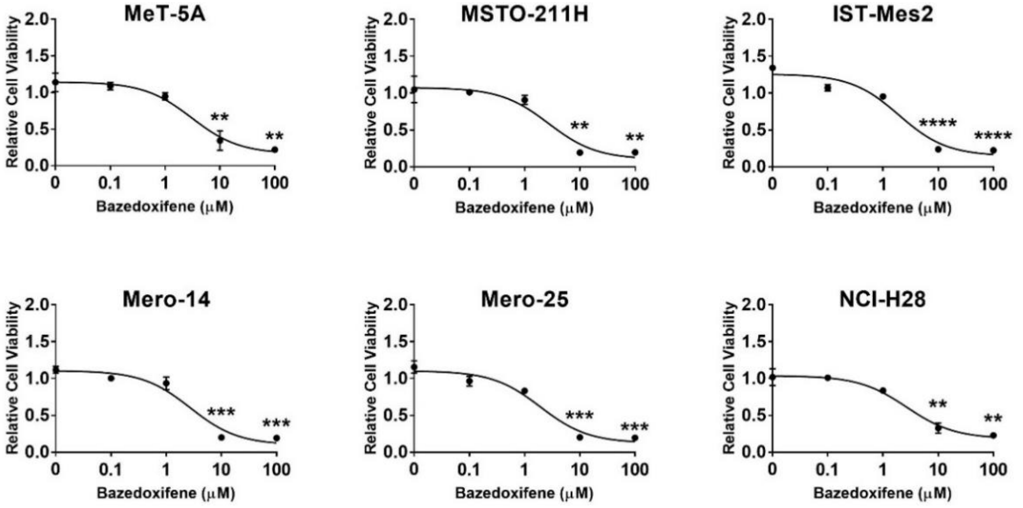

Bosutinib

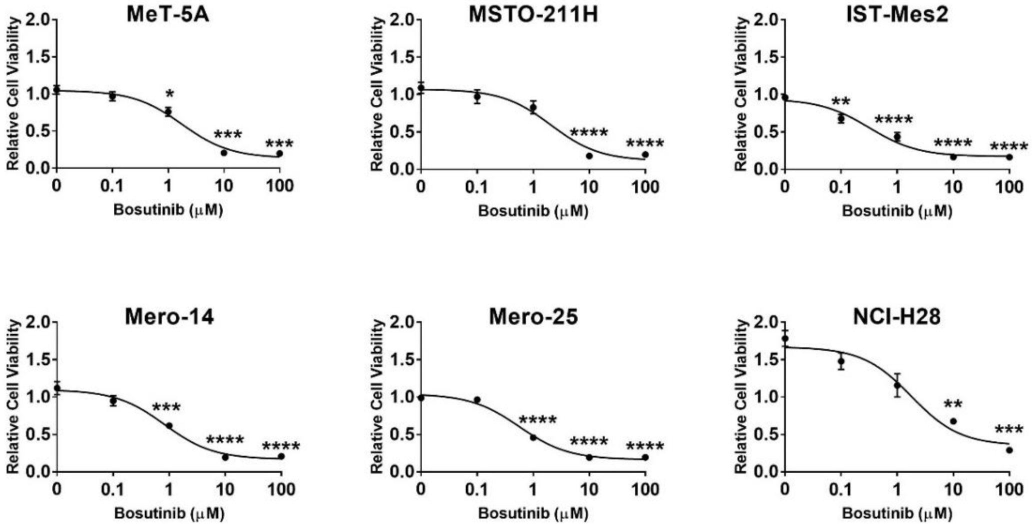

Carmofur

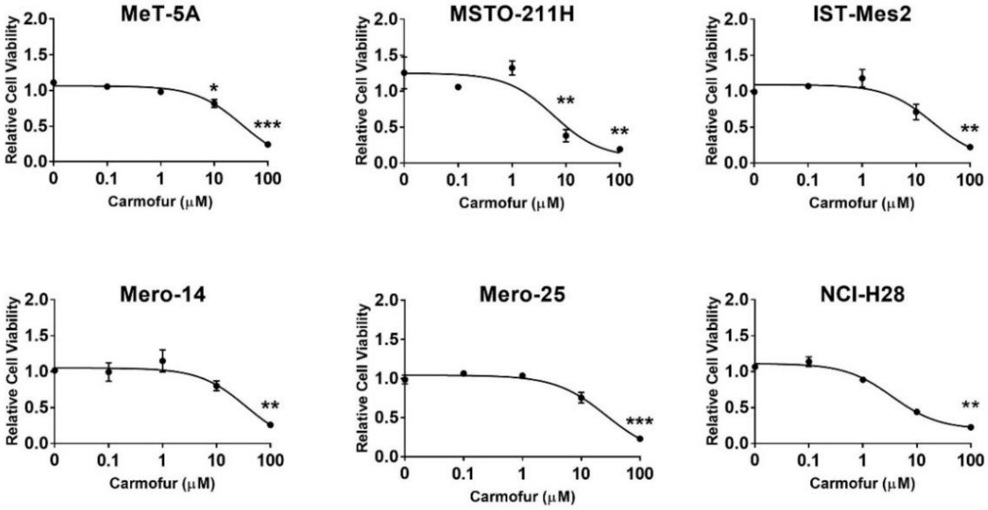

Cephalomannine

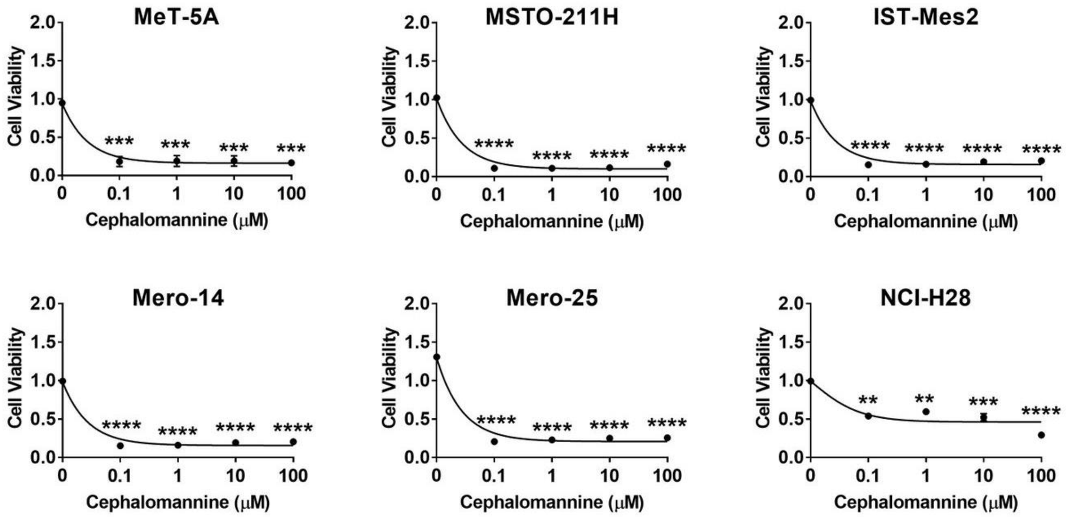

Cetrimonium bromide

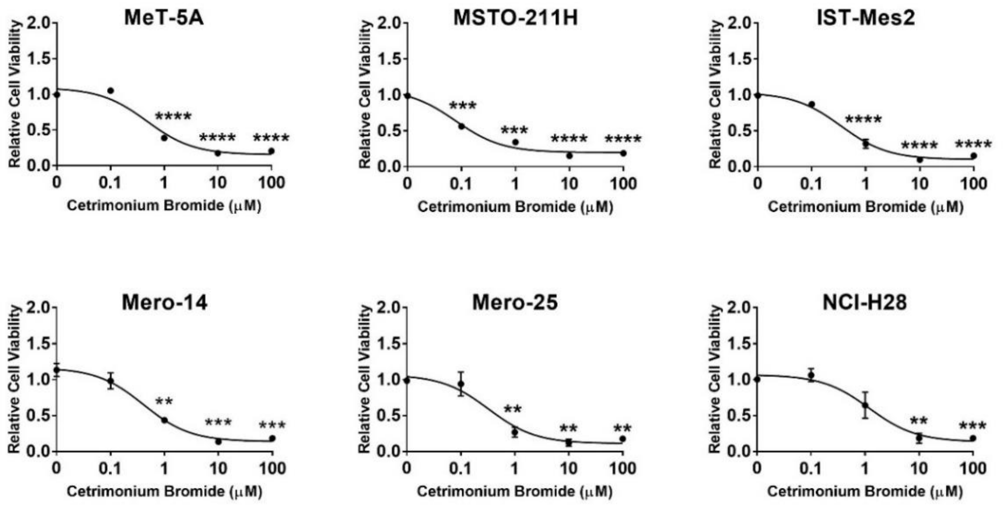

Ciclopirox

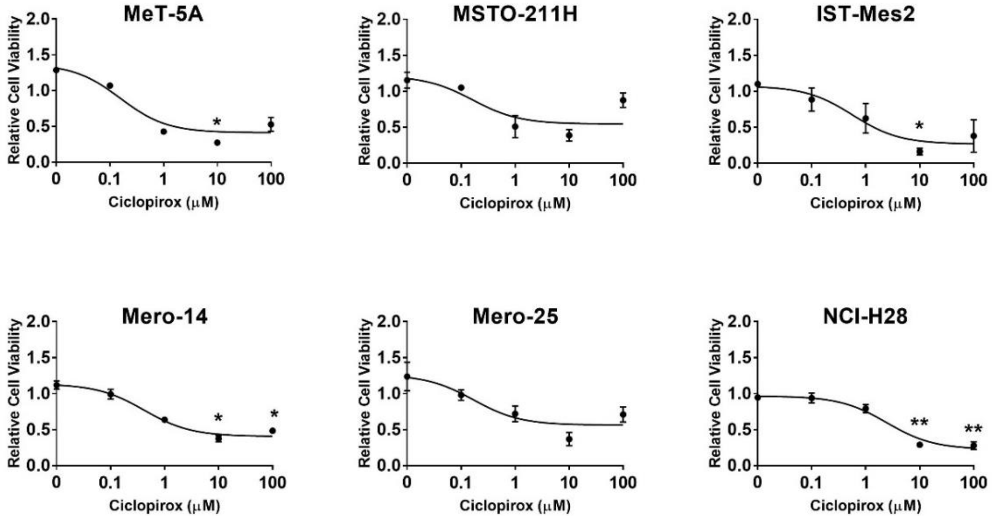

Cladribine

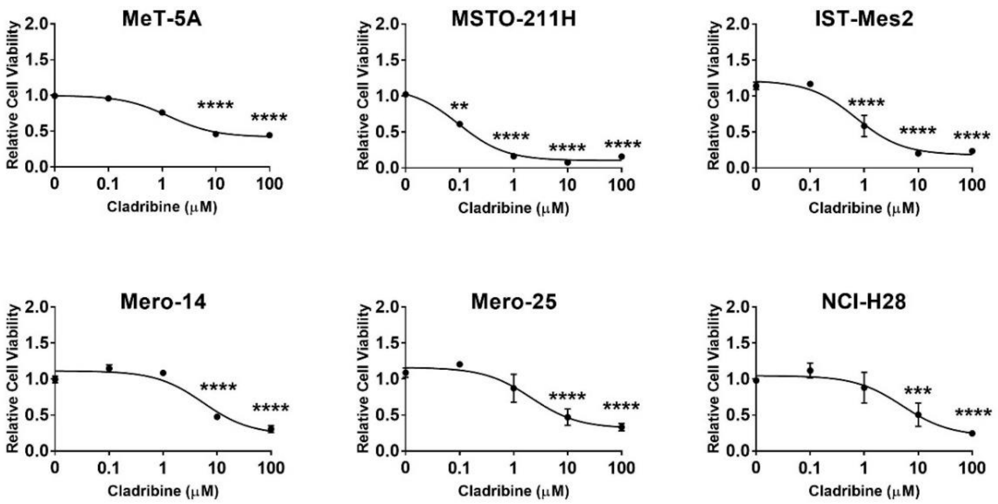

Clomifene citrate

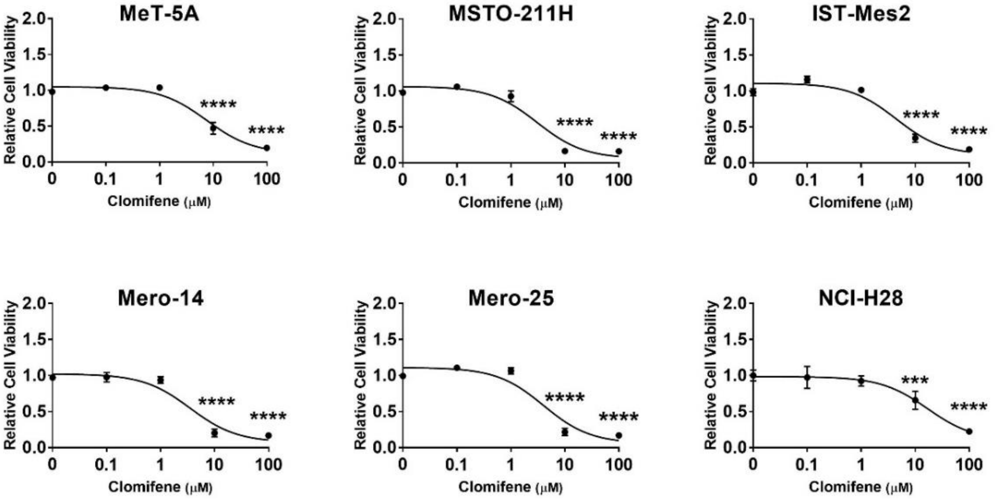

Cytarabine

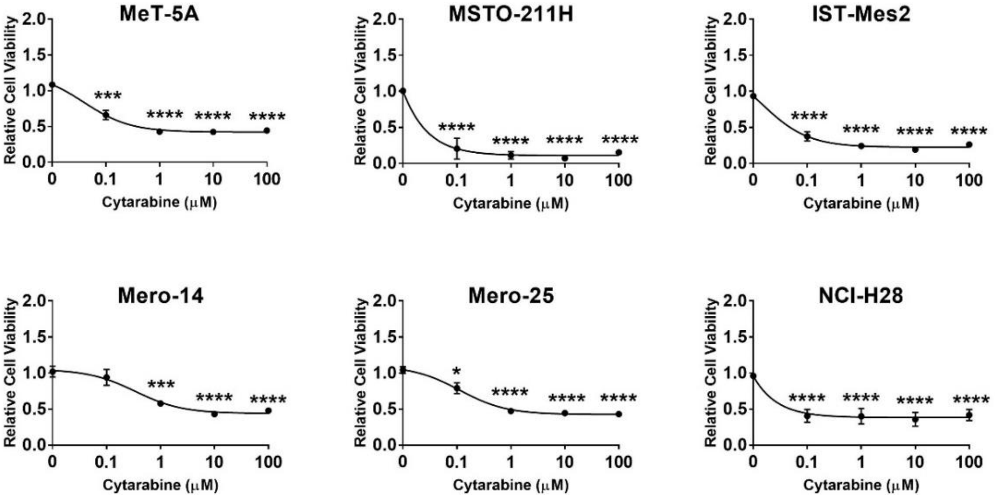

Digoxigenin

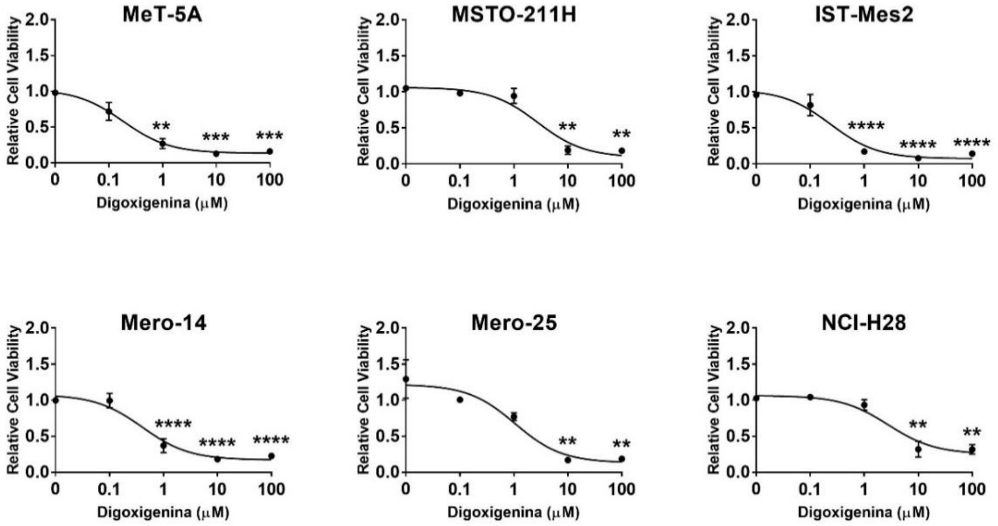

Dronedarone hydrochloride

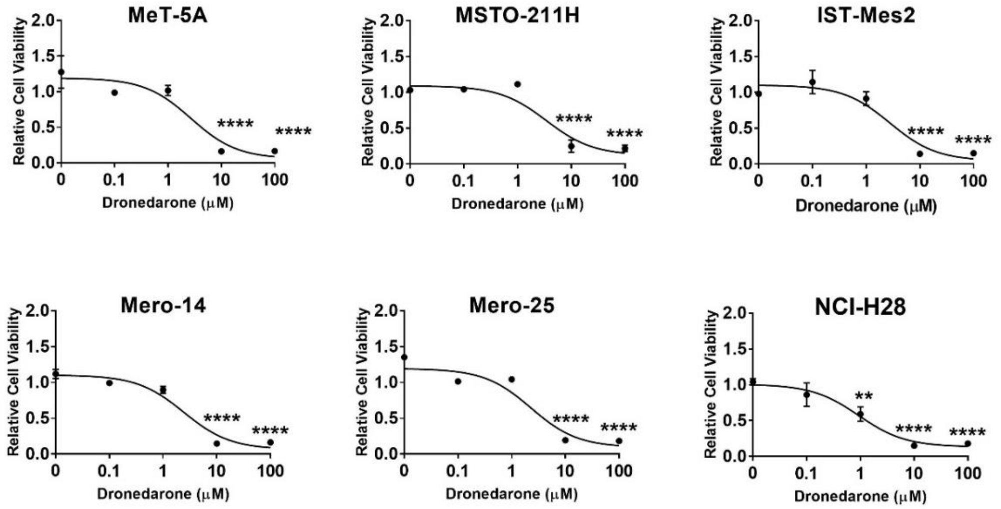

Emetine

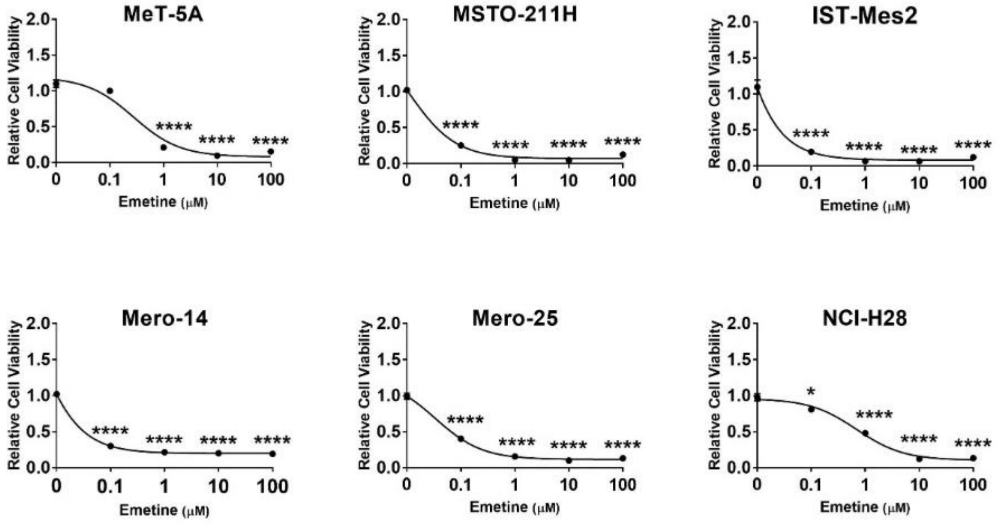

Fenbendazole

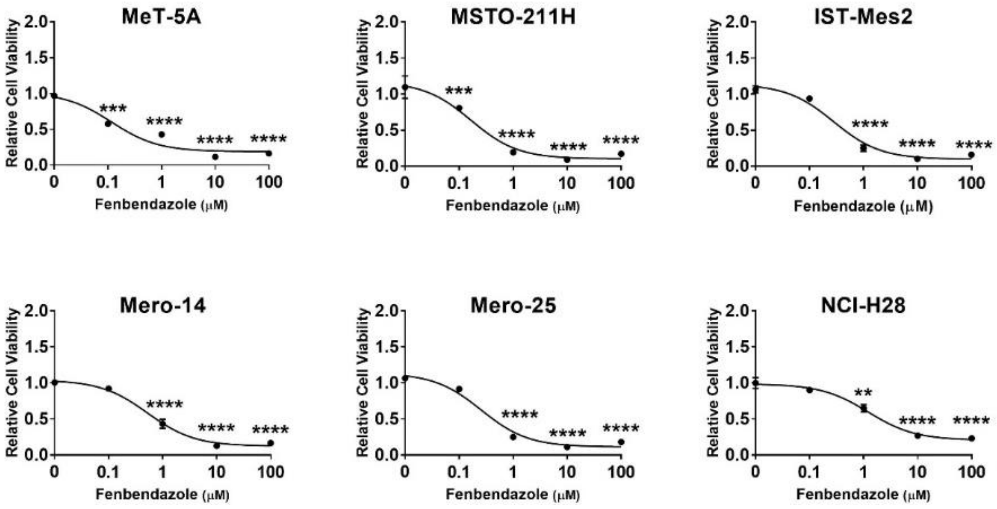

Flubendazole

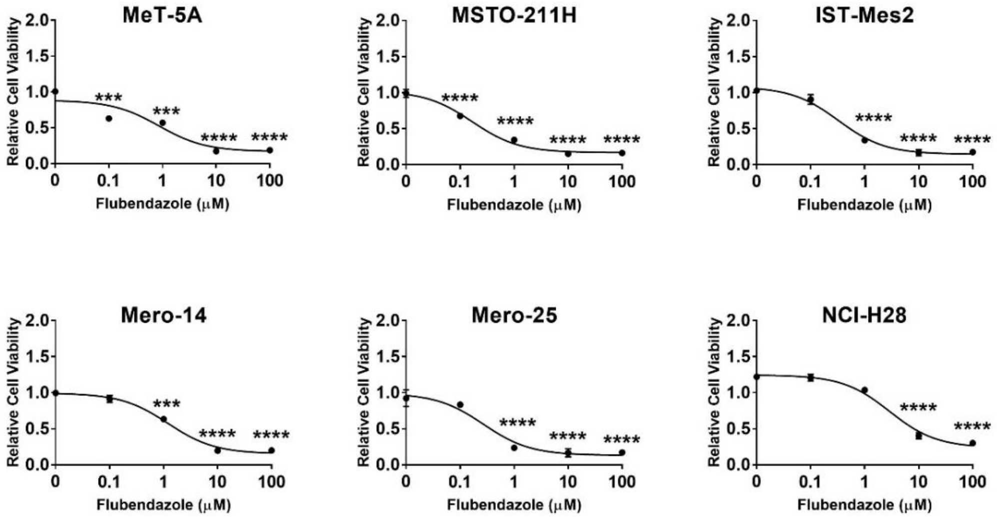

Gemcitabine hydrochloride

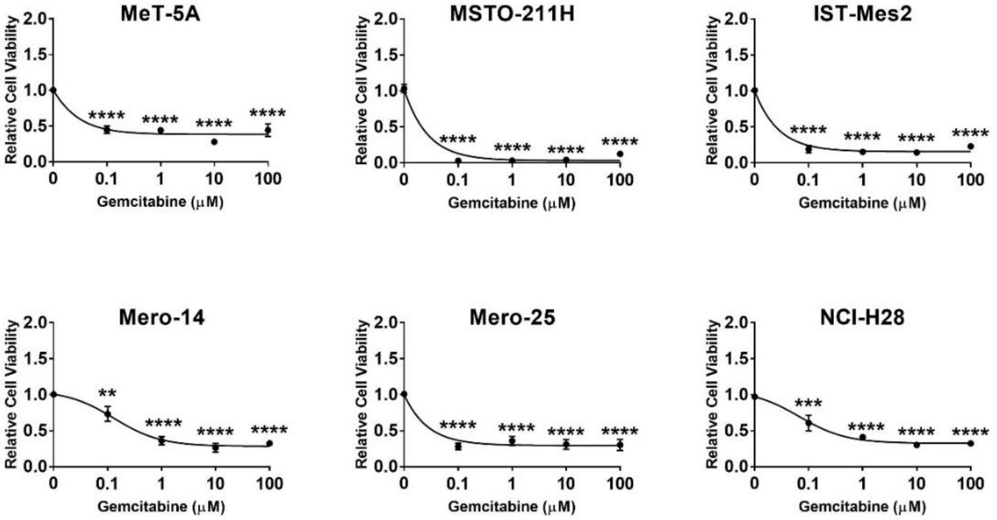

Miconazole

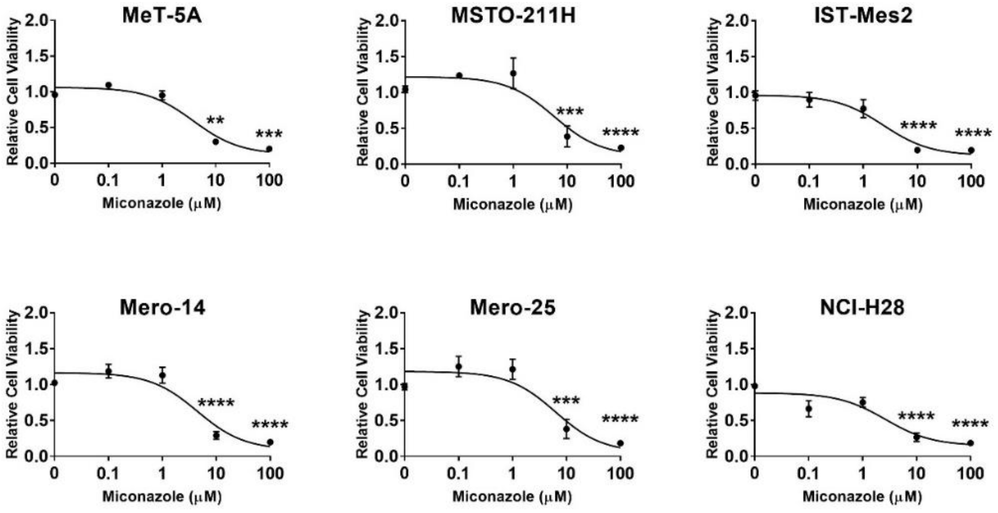

Niclosamide

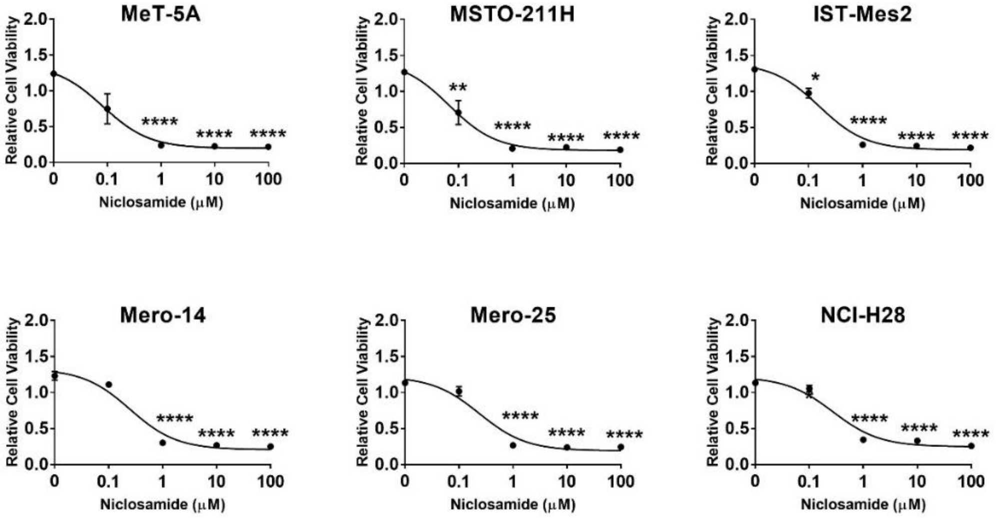

Ouabain

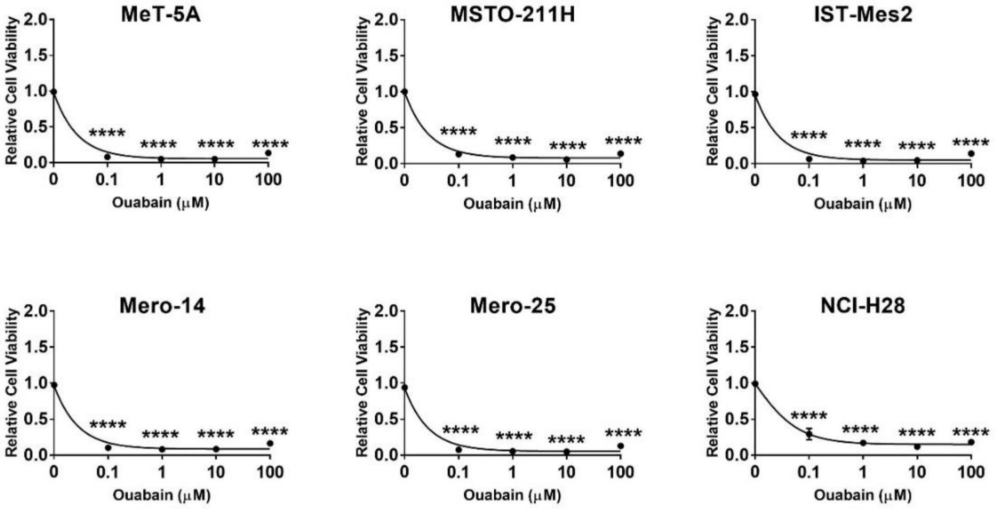

Oxethazaine

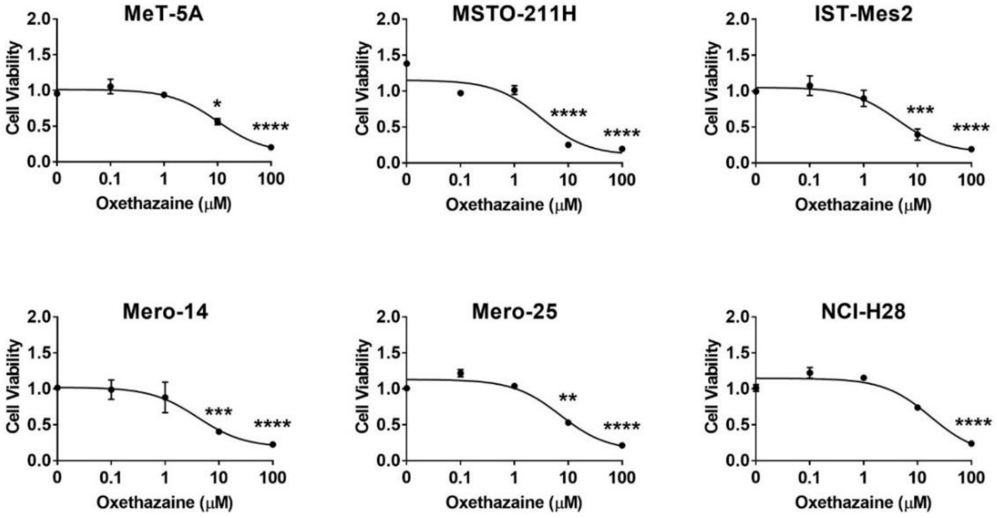

Penfluridol

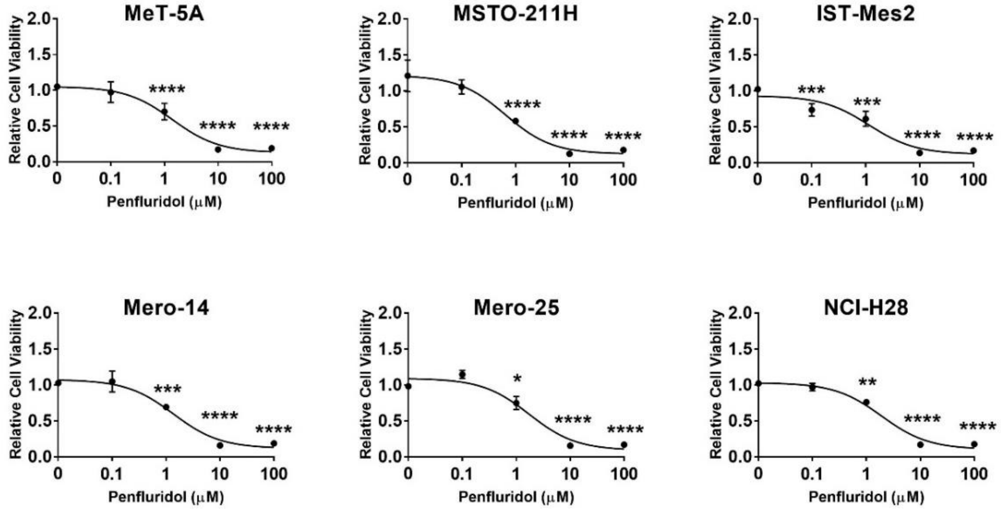

Pentamidine

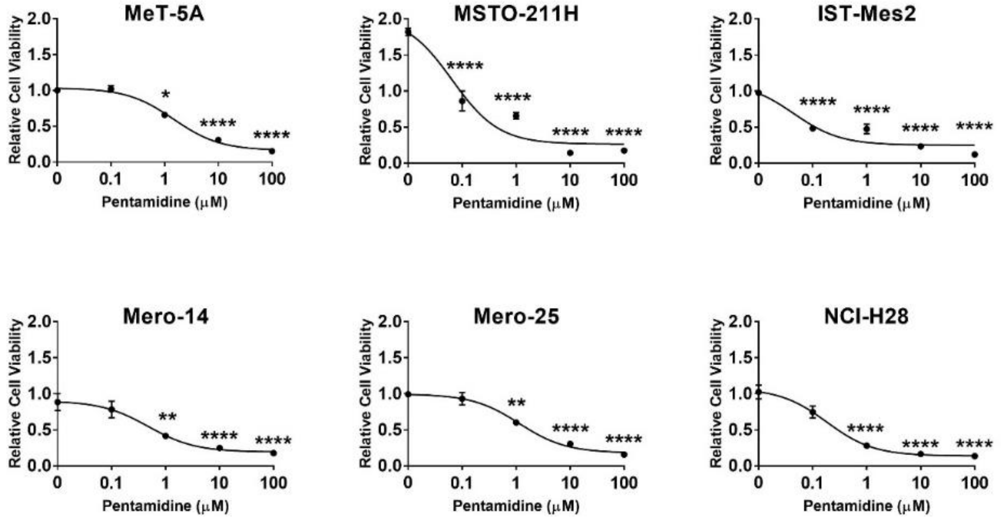

Pitavastatin calcium

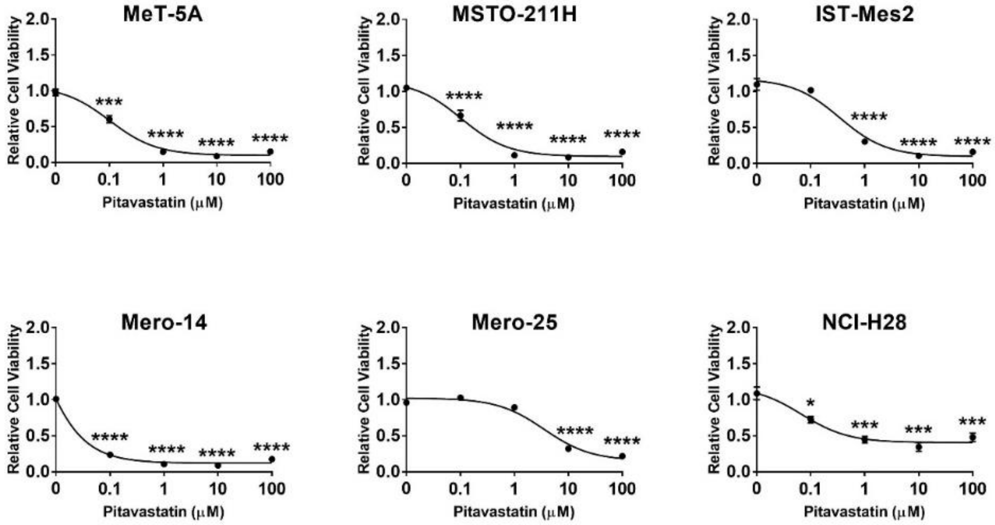

Pralatrexate

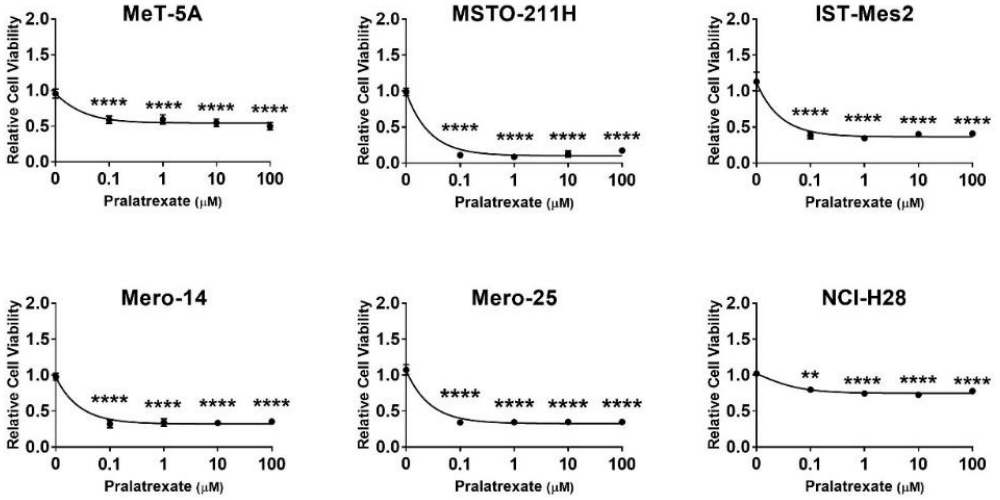

Pyrithione zinc

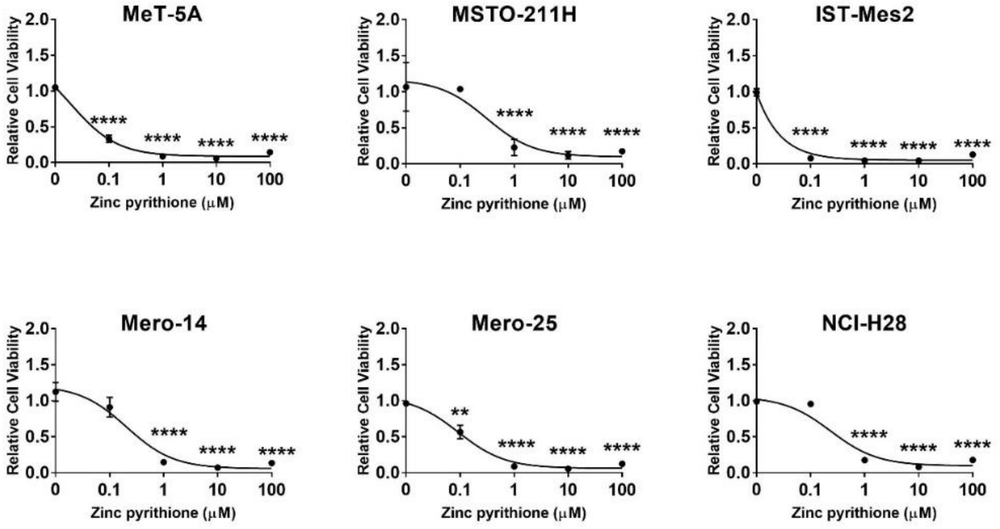

Sertraline hydrochloride

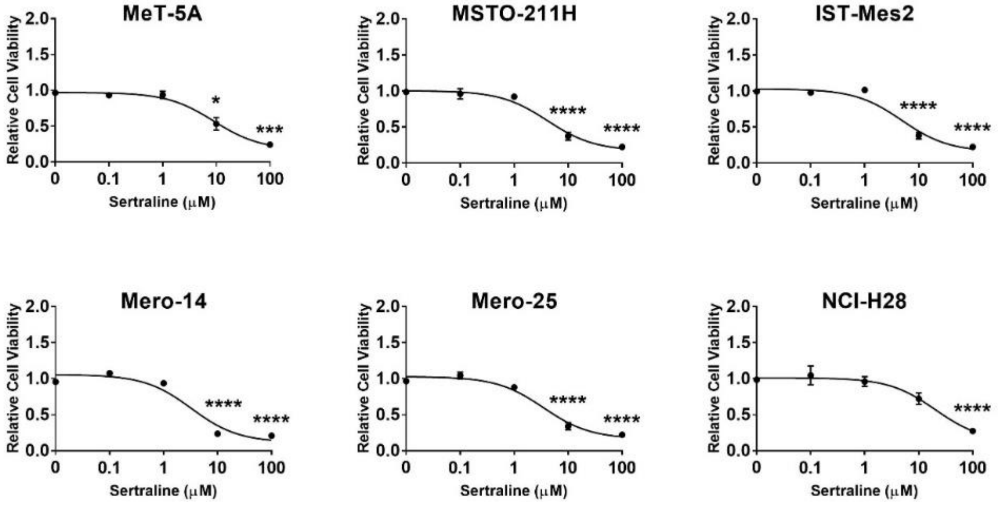

Solifenacin succinate

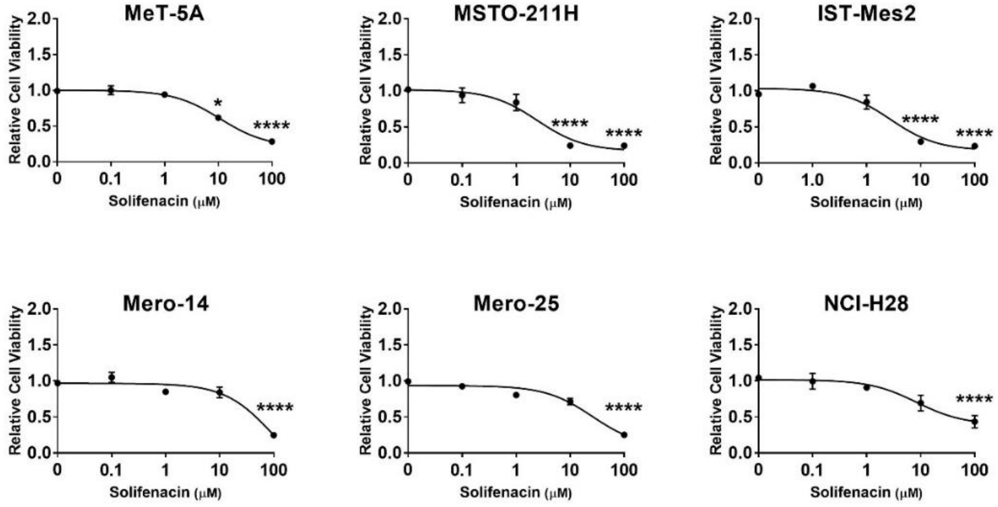

Sulconazole nitrate

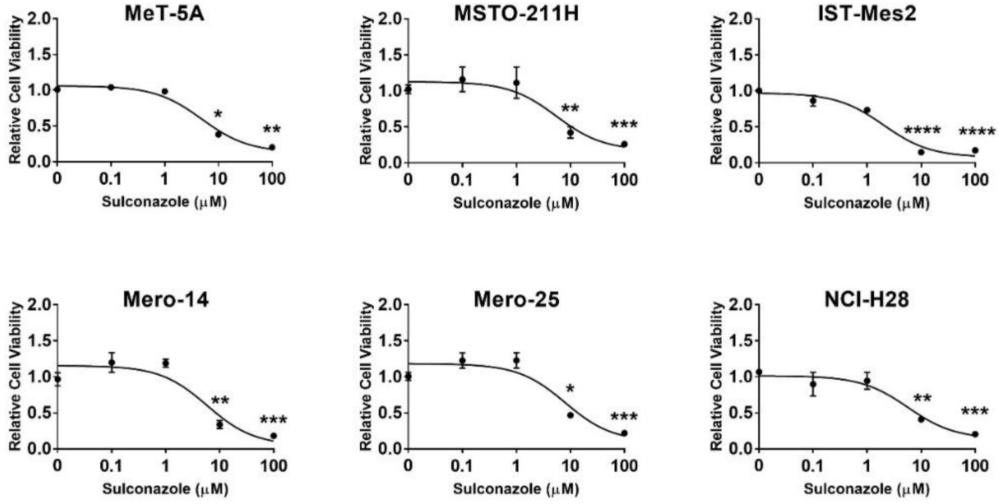

Tadalafil

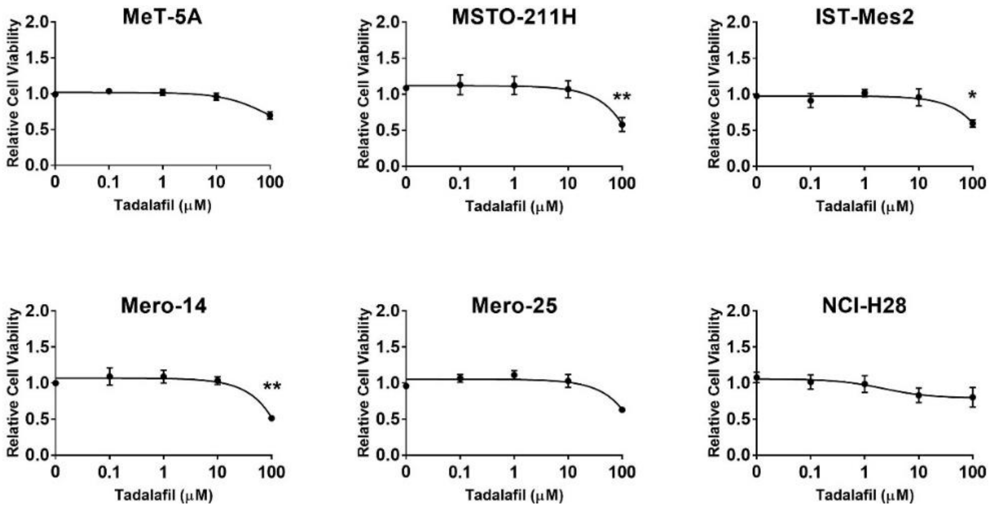

Terfenadine

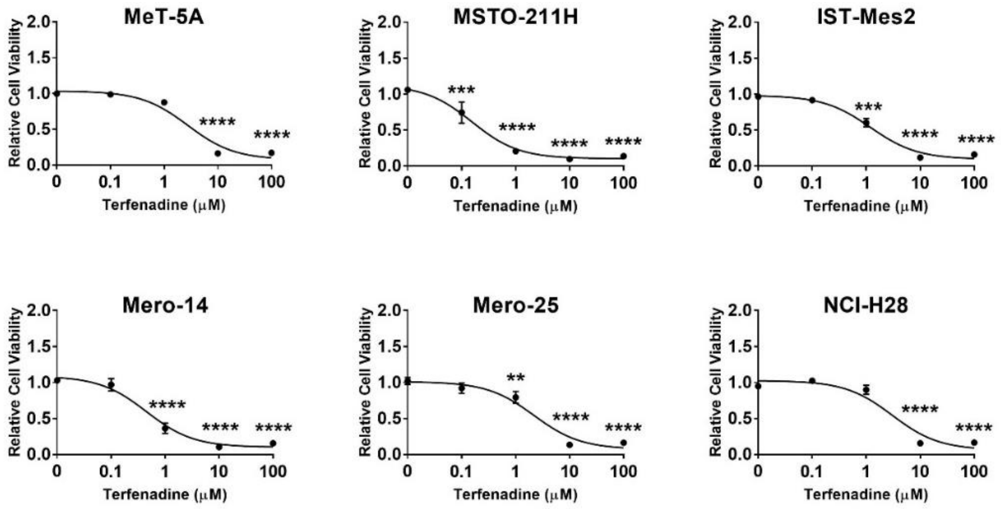

Thioguanine

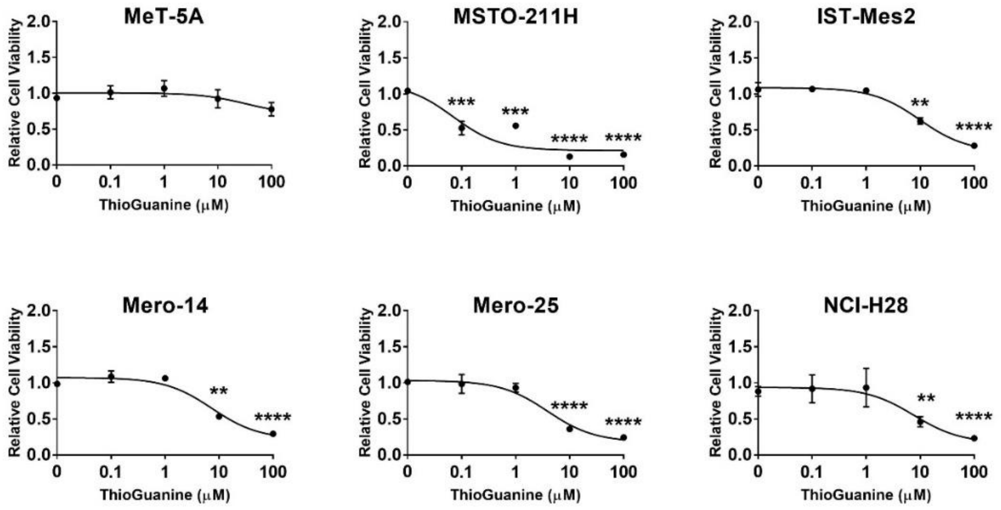

Thioridazine hydrochloride

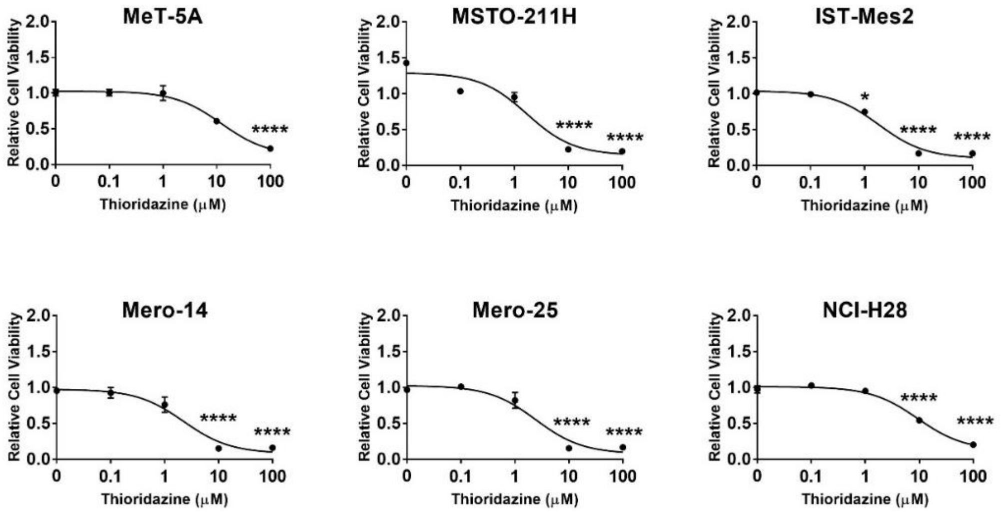

Thonzonium bromide

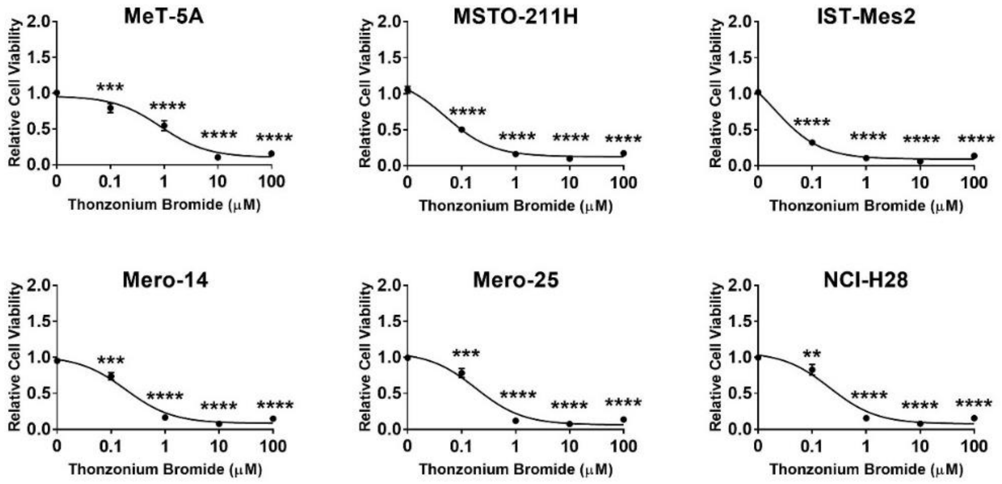

Tioconazole

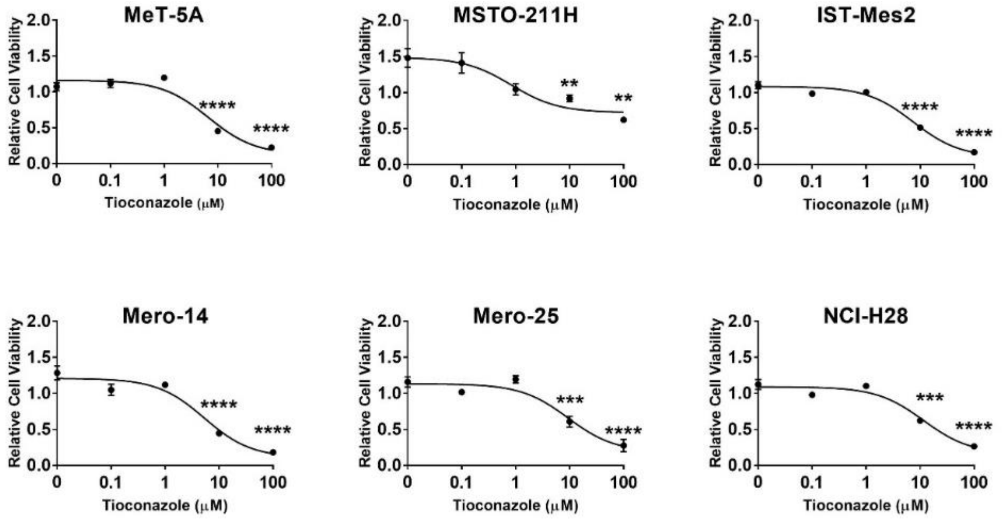

Toremifene citrate

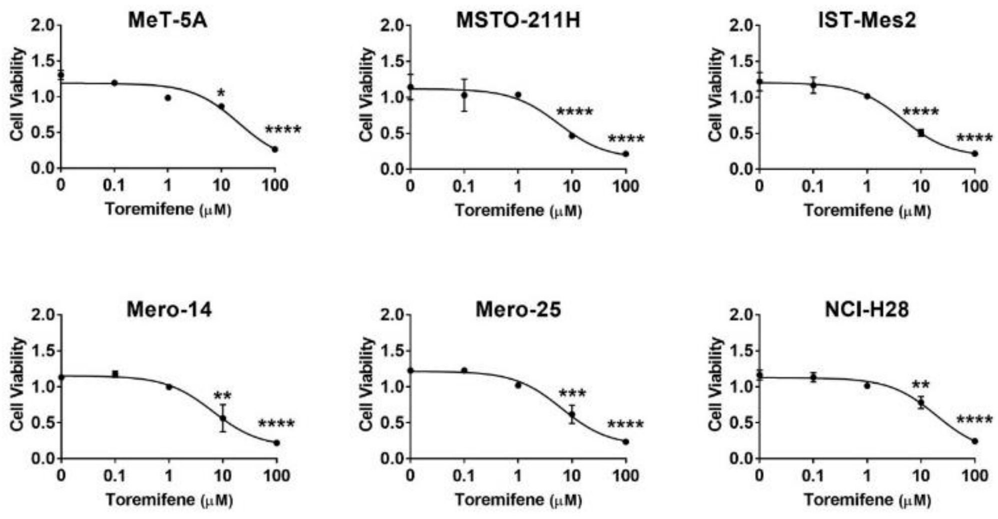

Trifluridine

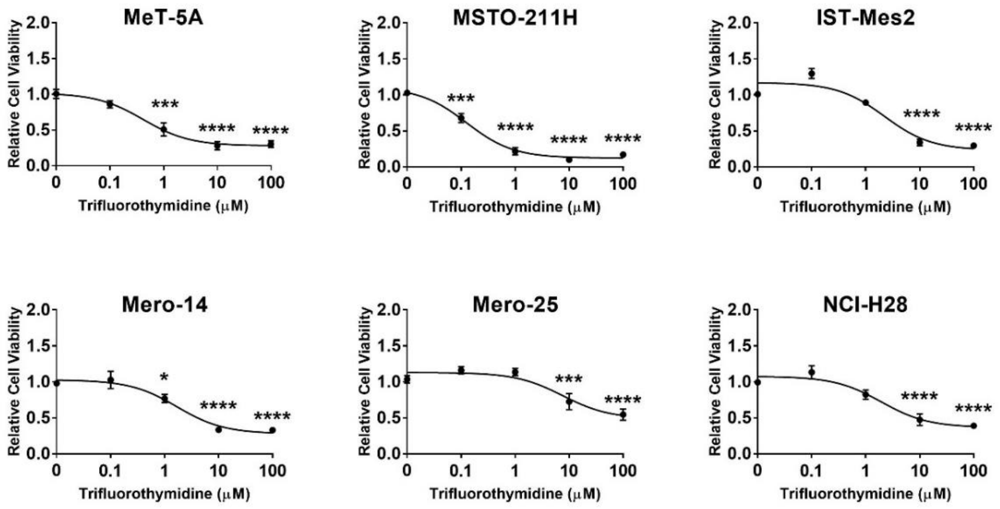

Vinorelbine

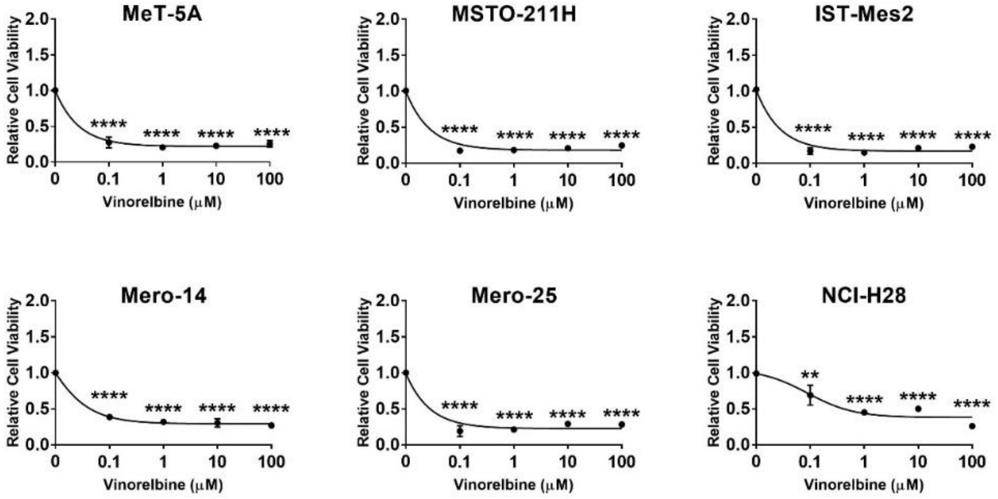

Supplement: Supplementary file 1 [file cancers-14-02527-s001.zip › cancers-1704721-Supplementary_dose-respoonse.pdf]
